# Mechanism of the Photochemical Isomerization and Oxidation of 2-Butenedial: A Theoretical Study

**DOI:** 10.3390/molecules28134994

**Published:** 2023-06-26

**Authors:** Andrea Maranzana, Glauco Tonachini

**Affiliations:** Dipartimento di Chimica, Università di Torino, Corso Massimo D’Azeglio 48, I-10125 Torino, Italy; glauco.tonachini@unito.it

**Keywords:** CASSCF, CCSD(T), DFT, 2-butenedial, tropospheric oxidation, reaction mechanism

## Abstract

Under tropospheric conditions, 2-butenedial is photochemically removed to produce secondary organic aerosol. Upon solar irradiation in the lower troposphere, the main photochemical products are ketene-enol (a key intermediate product), furanones, and maleic anhydride. The oxidative reaction mechanism was studied using the multireference method CASSCF to explore the hypersurface of the two most accessible singlet excited states, and by DFT for the ground state. Photoisomerization of 2-butenedial in the first excited state directly produces ground state ketene-enol upon nonradiative relaxation. From this intermediate, furan-2-ol and successively 3H-furan-2-one and 5H-furan-2-one are formed. The cooperative effect of two water molecules is essential to catalyze the cyclization of ketene-enol to furan-2-ol, followed by hydrogen transfers to furanones. Two water molecules are also necessary to form maleic anhydride from furan-2-ol. For this last reaction, in which one extra oxygen must be acquired, we hypothesize a mechanism with singlet oxygen as the oxidant.

## 1. Introduction

Unsaturated 1,4-dicarbonyl compounds are produced by an OH reaction with aromatic hydrocarbons, such as toluene, xylenes, trimethylbenzenes, ethylbenzene [1], and *o*-ethyltoluene, benzene [2], or by biomass combustion [3]. The oxidation mechanisms of some of these reactions have been theoretically investigated and have been shown to form carbonyl compounds (see for instance Ref. [4]). In particular, under atmospheric conditions, dicarbonyl compounds are rapidly photochemically degraded [5].

The photolysis and the reaction with OH of Z- and E,E-2,4-hexadienedial were investigated by Barnes et al. [6]. They concluded that the reaction with OH radicals was normally a secondary channel and only during the summertime could OH radical concentrations compete with photolysis. The identified products of the photolysis were 2-formyl-2*H*-pyran, 3,4-diformyl-cyclobutene, and 2-butenal-4-yl-ketene.

In a recent experimental study [7], Newland and co-workers investigated the photochemistry of 2-butenedial and 4-oxo-2-pentenal in an outdoor photoreactor (Euphore), in the presence and absence of OH radicals. The major products of the degradation of 2-butenedial, detected in situ by FTIR spectroscopy, were 3H-furan-2-one, maleic anhydride, CO, and an unidentified carbonyl compound. Maleic anhydride was supposed to form after tautomerization of the ketene-enol (which appears to be an important intermediate) to ketene-carbonyl. Formaldehyde, glyoxal, 5H-furan-2-one, and acrolein were also detected in minor quantity. The amount of HCO and CH_3_CO radicals were negligible under these experimental conditions. On the other hand, the formation of CO, CO_2_, and C_2_H_2_ products upon photolysis of butenedial was also reported by Marshall et al. and by Back and Parsons [8,9]. Maleic anhydride can in turn produce maleic acid. Röhrl et al. observed that its hydrolysis, promoted by water molecules, could be a possible source of the acid being detected in the atmosphere [10].

Tang and Zhu photolyzed butenedial at 193, 248, 280, 308, 351, 400, and 450 nm, and acrolein and 3H-furan-2-one were detected [11]. They did not detect HCO radicals in the region 280–450 nm. In a previous study by Bierbach et al., 3H-furan-2-one and maleic anhydride were also detected by FTIR spectra, after irradiation at 320 < λ < 480 nm [12]. They also hypothesized a reaction mechanism for 3H-furan-2-one formation via a diradical or zwitterion intermediate. A ketene-enol was identified as an intermediate of the reaction and its production stopped when the irradiation was suspended. This ketene was postulated to form by a Norrish type II process [13,14], from 2-butenedial. Intramolecular H-atom transfer leading to a ketene was also observed under photolysis of other ketones [15]. Interestingly, Newland et al. observed that the ketene-enol was formed when 2-butenedial was irradiated by solar light, and rapidly disappeared in the dark, and its depletion was related to the formation of furanones and maleic anhydride (Figure S10). The authors hypothesized that 3H-furan-2-one was formed by cyclization of the ketene-enol [7]. Such a reaction was also observed by other authors in studies of the aromatic compound phthalaldehyde [16,17,18,19]. When *o*-phthalaldehyde was irradiated with UV light, phthalide and a dimeric product were detected. Scaiano et al. concluded that a biradical with a short lifetime (1.6 μs) was produced by an intramolecular hydrogen transfer, and the reaction did not involve triplet states because they had a longer lifetime. They proposed a ketene-enol and a cyclic enol as intermediates [16]. The phototautomerization of *o*-phthalaldehyde was studied by multireference calculations by Blancafort et al. [17]. Their results showed that ketene-enol is formed by H transfer in the S_1_ state, then a conical intersection S_1_/S_0_ led to the intermediate in the ground state. The authors did not consider the triplet states. Fröbel et al. [18] have used the femtosecond-stimulated Raman spectroscopy and quantum calculations to study of phthalide formation from *o*-phthalaldehyde. The authors also identified the ketene-enol as the intermediate of reaction, formed by relaxation from the lowest excited singlet state to the ground state. However, ISC (S_1_/T_1_) seemed to be very efficient (≈5 ps), and they concluded that the triplet path could also be relevant.

He et al. [20] studied the photochemistry of butyrophenone using a complete-active-space self-consistent field (CASSCF) and density functional theory. The authors located all the minima, TS, and minimum energy crossing points among the S_1_, T_1_, and T_2_ states. They concluded that the S_1_/T_1_ ISC occurred at a low rate; however, the S_1_/T_2_ ISC was a fast process, and the T_2_/T_1_ internal conversion was expected to be extremely fast. The overall process was fast S_1_/T_1_ conversion (approximately 10^11^ s^−1^). The 1,5-H shift (Norrish type II process) occurred in the T_1_ state and led to a triplet 1,4-biradical intermediate.

Rowell et al. [21,22] studied 20 carbonyl compounds, among which 2-butenedial was not included. The photochemical process can occur in T_1_ or S_1_ states, depending on the reactants, namely on the S_1_ energy threshold for the Norrish type II reaction. When the S_1_ threshold is high, the T_1_ state becomes dominant. In the case of some α,β-unsaturated carbonyls, they concluded that the photoisomerization occurs in the S_1_ state and then crosses to S_0_ via the S_1_/S_0_ conical intersection. When the S_1_ energy barrier is high, an ISC to T_1_ transition occurs. With saturated carbonyls, photolysis in the T_1_ state is competitive or dominant. The α-bond cleavage photolysis of 20 atmospherically relevant carbonyls is possible in T_1_ state or on internally hot S_0_ [23].

On the other hand, Liu et al. observed that, when the reaction of butenedial was initiated by OH radicals, the products detected by gas chromatography/ion trap mass spectrometry analysis were formaldehyde, acrolein, glycolaldehyde, glyoxal, and malonaldehyde [24]. A hypothesis regarding the possible reaction mechanisms of these products was proposed.

The rate constant for the reaction *E*-butenedial + OH was assessed by Martín et al. as 3.45 ± 0.34·10^−11^ molec cm^3^ s^−1^, mainly due to the H abstraction process, whereas the photolytic rate coefficient was 3.6 ± 0.03·10^−4^ s^−1^. Therefore, under typical atmospheric conditions, the photolysis reaction is considered the major atmospheric sink for E-butenedial [25].

Despite all these studies, the formation mechanism of 3*H*-furan-2-one and maleic anhydride under photolysis of butenedial remain still uncertain and open to further investigations.

In this study, we theoretically investigate the singlet reaction mechanisms of 2-butenedial photooxidation, focusing on the formation of the experimentally detected furanones (isomerization) and maleic anhydride (oxidation). The postulated formation mechanisms of these products proposed in the experimental papers will be flanked by our computations, by which we will explore the lowest energy singlet surfaces to see if some viable pathways to these products are present; if these pathways are fully defined on the excited surfaces or only in part; in this case, we will determine under which circumstances the ground state surface is involved.

Because we found a low-barrier mechanism leading to the ketene-enol in the S_1_ surface, followed by an S_1_/S_0_ conical intersection, we focused on the singlet multiplicity, but we cannot exclude the contribution of low-energy triplet states.

**Figure 1:**
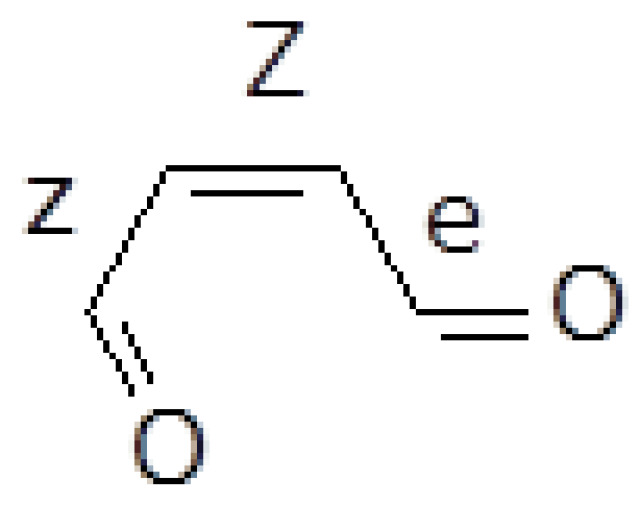
Zze butenedial.

The more promising reactant isomer to initiate some chemical transformation is the (Z)-2-butenedial with the two carbonyls in *s-cis* and *s-trans* conformations with respect to the central double bond (for short: Zze, in Figure 1). 

## 2. Results

### 2.1. Formation of Ketene-Enol

**Scheme 1:**
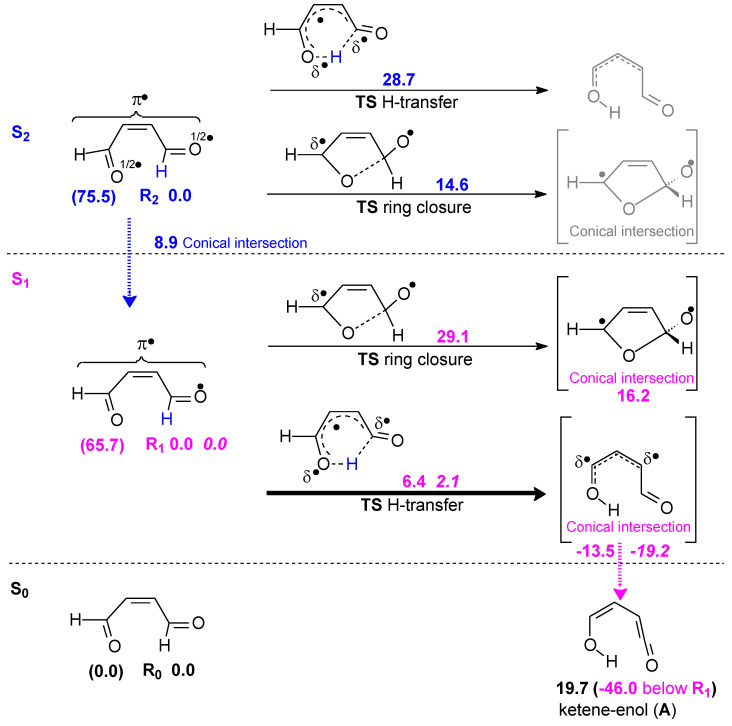
Photochemical formation of ketene-enol. Potential energies in kcal mol^−1^, calculated at the SA-CASSCF(12,10)/6-31G(d) level of theory. SA-CASSCF(12,10)/cc-pVTZ//CASSCF (12,10)/6-31G(d) energies in italics. Left: adiabatic transition energies are shown in parentheses.

In 2-butenedial, the first two excited states S_1_ and S_2_ are close in energy: with respect to the ground state, 82.2 and 88.9 kcal mol^−1^ (corresponding to ≈350 nm, S_1_ and ≈320 nm, S_2_, respectively) for vertical transitions, or 65.7 (S_1_) and 75.5 (S_2_) kcal mol^−1^ for adiabatic transitions, as presented in Scheme 1. Ketene-enol is formed by the photochemical process. When irradiated, aldehydes with chains longer than four carbon atoms undergo a Norrish type II reaction step, which involves intramolecular H atom abstraction in the first excited state. The two closest excited states are both characterized by excitation from the in-plane p_O_ − p_O_ and p_O_ + p_O_ combinations to the empty π^4^ orbital (see Section 3).

In the S_2_ state, the barrier for hydrogen transfer from C to O to obtain ketene-enol is very large (ΔE^‡^ = 28.7 kcal mol^−1^). Ring closure (ΔE^‡^ = 14.6 kcal mol^−1^) has a lower barrier, but internal conversion through a conical intersection S_2_/S_1_ is the fastest step (ΔE^‡^ = 8.9 kcal mol^−1^). In any case, the oscillator strength (Table A1 in the Appendix A) indicates that the S_2_ state is scarcely populated.

In the S_1_ state, the H transfer has a barrier of 6.4 kcal mol^−1^ only, much lower than ring closure (ΔE^‡^ = 29.1 kcal mol^−1^). However, the ketene-enol in the first excited state does not exist as a minimum, since a ketene-enol-like structure corresponds to a conical intersection S_1_/S_0_. A second conical intersection with geometry closer to the TS for H-transfer is present but its energy is less favorable. The energies of the most relevant pathway (thick arrows in Scheme 1) were refined by calculations with a better basis set (see Section 3). With the larger basis set, the Norrish type II barrier is only ΔE^‡^ = 2.1 kcal mol^−1^. The atomic displacements for the imaginary frequency of the TS H-transfer (1292 i cm^−1^) correspond to a [1,5] hydrogen shift. This barrier was also estimated by EOM-CCSD/6-31G(d) computations [26,27,28], which gave an even lower value ΔE^‡^ = 0.1 kcal mol^−1^.

The non-existence of ketene-enol as a minimum in the S_1_ hypersurface and the conical intersection S_1_/S_0_ was also found by Blancafort for *o*-phthalaldehyde photochemistry [17].

### 2.2. Formation of Furanones

Considering the work of Newland et al. [7], it can be seen in Figure S10 that during irradiation, the formation rates of 2-butenedial and ketene-enol showed specular behavior: as butenedial was photolyzed and its concentration declined, that of ketene-enol increased. In the dark, a similar specular behavior was observed for ketene-enol and 3H-furan-2-one. It can be noticed that 3H-furan-2-one is formed both during irradiation and in the dark. When irradiated, its concentration increased almost linearly; in the dark, its formation went on, but slowing down and tending to a plateau. This behavior can be attributed to the conversion of the declining amount of ketene-enol to 3H-furan-2-one. Our proposed mechanism is consistent with this picture.

**Scheme 2:**
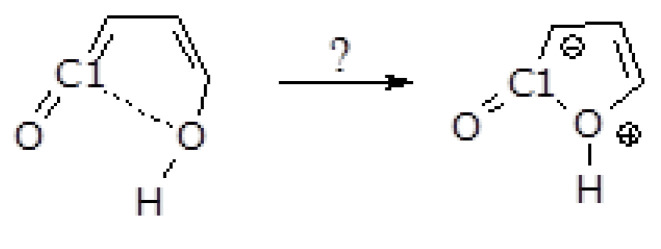
Conceivable ketene-enol to furanone ring closure step.

Ring closure on the ground state surface to form the furanone ring directly from the ketene-enol (**A**) is not possible (see Scheme 2). When the enolic oxygen approaches C1, a purely repulsive energy profile is obtained. The hypothetically zwitterionic intermediate, the precursor of the furanones does not even exist as a minimum on the potential energy surface (compare Figure 6 in Ref. [12] and Figure 9 in Ref. [7]).

Despite our efforts to identify a viable pathway connecting ketene-enol **A** to 3H-furan-2-one and 5H-furan-2-one, all attempts failed. Further details are provided in the Scheme S1 in the Supplementary Materials.

Weingart et al. studied the formation of 3-methylphthalide from a ketene intermediate [29]. In their work, the ketene was formed by photoexcitation of *o*-acetylbenzaldehyde by a Norrish type II reaction, similar to that shown in Scheme 1. Their computed barrier connecting the ketene-enol to a cyclic enol, the precursor of the lactone, was 40 kcal mol^−1^. The authors demonstrated that water molecules catalyze ring closures. Although a single water molecule was not sufficient to significantly change the energy barrier, the addition of a second water molecule lowered the above-mentioned barrier to 2.5 kcal mol^−1^. In effect, a cooperative action of water has been invoked under many disparate circumstances (see for instance Refs [30,31]).

In our case, the formation of the main products requires the presence of water molecules too, and a cooperative effect seems to be needed to promote both cyclization and hydrogen migration to form furan-2-ol and the two furanones. An intermediate similar to furan-2-ol was also previously hypothesized by Scaiano et al. [16] and Fröbel et al. [18], who both studied the photochemistry of *o*-phthalaldehyde.

Under typical tropospheric conditions, with a relative humidity of 50%, the water concentration in the gas phase is about 10^17^ molec cm^−3^, but in the Euphore photoreactor, Newland et al. carried out their experiments under dry conditions [7]. The water vapor concentration was estimated to be below 1% [32] but above 1·10^15^ molec cm^−3^. Under such conditions, the ratio of water to 2-butenedial molecules is still above 100:1, and the assumption of water intervention in the reaction mechanism seems to be reasonable.

**Scheme 3:**
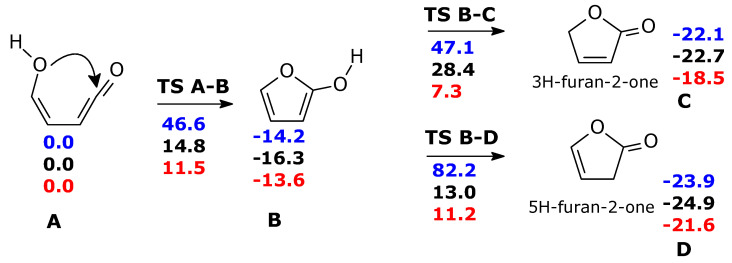
Formation of furanones form ketene-enol, in the ground state. Blue: gas phase without explicit water molecules; black: gas phase with one explicit water molecule; red: gas phase with two explicit water molecules. For the sake of clarity, the two water molecules are not shown in this scheme, but the relevant transition structures are shown in Figure 2. ΔG(298 K) in kcal mol^−1^.

Without water molecules, the barriers for ketene-enol cyclization (**TS A-B,** ΔG^‡^ = 46 kcal mol^−1^) and tautomerization of furan-2-ol (ΔG^‡^ = 61.3 and 96.4 kcal mol^−1^ for **TS B-C** and **TS B-D,** respectively) are exceedingly large (Scheme 3, in blue). The second water molecule is particularly important for **TS B-C**. Through water intervention, cyclization through **TS A-B** becomes viable. In **TS A-B**, the imaginary frequency (164 i cm^−1^) corresponds to multiple H transfers, which ultimately results in an H shift from one oxygen (enole) to the other (ketene) (see Figure S2).

**Figure 2:**
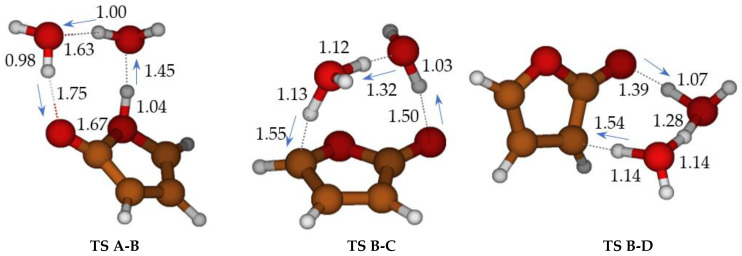
Cyclization of ketene-enol **TS A-B**, hydrogen migrations **TS B-C,** and **TS B-D** catalyzed by two water molecules. Bond distances in Ångstrom.

The free energy barriers for the formation of 3H-furan-2-one (**TS B-C**) and 5H-furan-2-one (**TS B-D**) in the presence of two water molecules were significantly lowered. Nevertheless, they remained still higher than the first step: (ΔG^‡^ = 20.9 kcal mol^−1^ for the main product **C**, 24.8 kcal mol^−1^ for product **D**) (We have attempted to refine the free energy differences for **TS A-B**, **TS C-D**, **TS B-D**, at CCSD(T)/cc-pVTZ//M06-2X/cc-pVTZ level (with thermochemical correction at DFT level). ΔG = 11.4 (TS A-B), −13.5 (**B**), 10.0 (**TS B-C**), 14.6 (**TS B-D**) kcal mol^−1^, making reference to A) and 11.5 kcal mol^−1^ for **B**.

Further attempts to form furanones without explicit water molecules are reported in the Supplementary Materials.

### 2.3. Formation of Maleic Anhydride

Starting from the ketene-enol **A**, a widely accepted sequence of steps (triggered by OH and in the presence of a significant tropospheric NO_x_ concentration) could lead to maleic anhydride. The reaction begins with hydrogen abstraction by OH and concerted cyclization. Subsequent O_2_ addition, NO intervention to transform a peroxyl radical into an oxyl radical, and finally H abstraction by O_2_ would produce maleic anhydride with low-computed energy barriers (see Scheme S2 in the Supplementary Materials).

However, Newland et al. [7] experimented the addition of propan-2-ol as an OH scavenger and noted that the measured yield of maleic anhydride did not change significantly. This indicates that the above OH-initiated sequence, cannot be the dominant pathway for the anhydride.

**Scheme 4:**
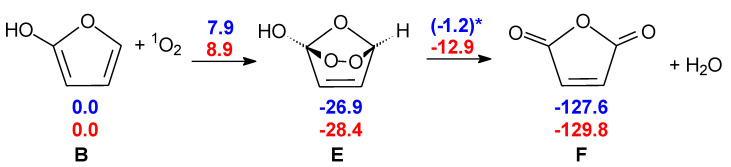
Formation of maleic anhydride from 2-furanol **B**, initiated by ^1^O_2_. Blue: gas phase without explicit water molecules; red: gas phase with two explicit water molecules. For the sake of clarity, the two water molecules are not shown in this scheme but a picture of **TS E-F** is shown in Figure 3. ΔG(298 K) in kcal mol^−1^. * This barrier does not directly lead to **F**: further steps are described in the Supplementary Materials (Scheme S3).

Consequently, we put forward a different mechanism in which another oxidant could be responsible for the initial step toward the anhydride. Based on its concentration and reactivity, singlet oxygen (^1^Δ_g_ O_2_, ^1^O_2_ for short) appears to be a reasonable candidate. ^1^O_2_ is detected in the troposphere at a concentration of ca. 10^8^ molec cm^−3^ [33], which is two orders of magnitude higher than that of the OH radical. ^1^O_2_ could be the initiator by adding to furan-2-ol **B**, which can form as seen in the presence of two water molecules (see Scheme 4).

**Figure 3:**
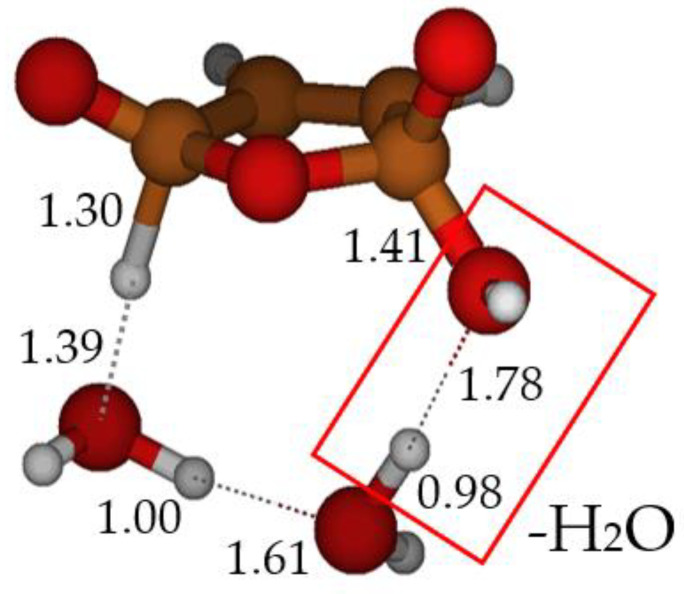
**TS E-F**: formation of maleic anhydride catalyzed by two water molecules. Bond distances in Ångstrom.

In our model, furan-2-ol **B**, formed after cyclization of the ketene-enol (see Scheme 1), undergoes a [π4 + π2] cycloaddition to form the endoperoxide **E**. The breaking of the O–O bond, accompanied by concerted H_2_O loss, leads to maleic anhydride **F**. The picture of this transition structure is shown in Figure 3. Although the O–O bond is weak, the free energy barrier for its homolytic dissociation is too large (25.7 kcal mol^−1^) compared to the experimental rate of the reaction. In this case, again, two explicit water molecules can catalyze the reaction, lowering the free energy barrier of **TS E-F** to 15.4 kcal mol^−1^. 

Other possible mechanisms of oxidation initiated by radical addition or hydrogen abstraction by OOH radical have been considered but they have been ruled out because of the high-energy barriers of some steps.

## 3. Materials and Methods

### 3.1. CASSCF Method

**Figure 4:**
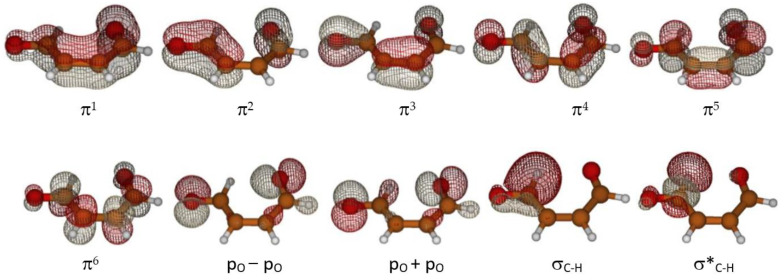
Active orbitals.

To explore the reaction hypersurface topology of the first and second excited states and formation of ground state ketene-enol, as described in Scheme 1, the multi-configuration self-consistent field approach (MCSCF), in its CASSCF (complete active space) version was used [34]. The third excited state lays about 100 kcal mol^−1^ (290 nm) above the ground state and its contribution should be negligible because of the scarce sunlight irradiation at ground level at that wavelength (see for instance Figure 3.32 ([35], p. 80)). The active space adopted in this study is labeled (12,10), indicating 12 electrons in 10 orbitals. It includes all six π system orbitals, two lone pairs of the oxygen atoms, and one σ_C–H_ bonding MO of the aldehyde with its antibonding counterpart (the C–H involved in the initial tautomerization step). A plot of the molecular orbitals in the active space is shown in Figure 4.

The excited state structures of the minima, TS, and conical intersections were calculated as the state average (SA-CASSCF) of the first three states, considering equal weights. In the multireference calculations, the basis set used to optimize the geometries and to assess the nature of the critical points was Pople’s polarized split valence shell 6-31G(d) [36,37]. For the S_1_ state only, onto which the initial important step takes place, the energies were refined by single point calculations with Dunning’s cc-pVTZ basis set [38].

The SA-CASSCF gradient optimizations with numerical Hessian computations were performed by the MOLPRO program [39]. Numerical harmonic vibrational frequencies were computed to test the nature of the critical points.

TD-DFT [40,41] and EOM-CCSD [26,27,28] were used to estimate the vertical transition energies and related oscillator strengths (see Appendix A).

### 3.2. Density Functional Theory Study

To study the main products formation pathways, since explicit water molecules proved necessary, the occurrence of different chemical events and ^1^O_2_ intervention had to be considered (see the Section 2). Consequently, the CASSCF active space would have grown too large. For this reason, the stationary points of chemical interest on the ground state energy hypersurface (minima and transition structures) were determined at DFT by gradient procedures and with the M06-2X functional [42] and the cc-pVTZ basis set [38]. The nature of the critical points was tested by vibrational analysis. Harmonic vibrational frequencies were computed by analytically determining the second derivatives of the Hessian matrix. The thermochemical corrections provided estimates of the relative Gibbs free energies (ΔG). Gibbs free energy and, in particular, the ΔS term were estimated by the total partition function, which includes translational, rotational, electronic, and vibrational contributions [43,44]. ΔG values at T = 298.15 K, are reported in this paper.

For singlet diradicaloid structures, originating from the reaction with ^1^O_2_, the wavefunction stability was checked. Upon relaxation, by allowing orbital rotations, the resulting spin-mixed wavefunction gives a better description of the electron distribution, but alters the energy, so the energy values were corrected by Yamaguchi’s formula [45,46].

Some energies were refined using the CCSD(T) [47,48] method with the cc-pVTZ basis set [38].

Geometry optimizations and thermochemical calculations at DFT were carried out by using the GAUSSIAN16 system of programs [49]. Figures have been obtained by the program MOLDEN [50].

## 4. Conclusions

From the experimental investigation by Newland et al. [7], we know that that the most important photochemical products obtained upon solar irradiation of butenedial are 3H-furan-2-one and maleic anhydride. Moreover, a key intermediate product is the ketene-eno, generated by H transfer in the irradiated Zze reactant, H moving from one aldehydic carbon to the opposite oxygen. This theoretical study aimed to elucidate the mechanism by which the main products are obtained upon solar irradiation in the lower troposphere.

Two principal conclusions are drawn from the scrutiny of the three lowest electronic states in Zze butenedial.

(1) The second excited state has a role only in populating the first excited state because the system easily passes through a conical intersection S_2_/S_1_, in correspondence with geometry very close to that of the reactant Zze butenedial itself. In the first excited state, an even lower barrier is found in the correspondence of H transfer towards ketene-enol, and isomerization takes place. However, no ketene-enol minimum is present on this excited state surface, instead a conical intersection S_1_/S_0_ is found just past the isomerization transition structure. Therefore, the reacting system is easily funneled to the ground state in correspondence of the ketene-enol minimum. Thus, although the reaction is triggered by light absorption, and the first isomerization step takes place involving the excited reactant, all the subsequent chemistry takes place in the ground state. 

(2) The free energy barriers that the system should overcome to form the main products appear to be too high, even considering the energy gain consequent to the decay to the ground state. Therefore, the presence of two cooperating water molecules proved necessary, more than convenient: they operate by passing one hydrogen from one water to the other, thereby allowing a hydrogen shift from two positions of the same intermediate. Namely, they first catalyze the hydrogen transfer necessary for the cyclization of ketene-enol to furan-2-ol. Then, from furan-2-ol, further water-mediated H transfers produce the two final furanones. On the other hand, to obtain maleic anhydride, one extra oxygen is incorporated into the molecule. Because the findings of Newland et al. tend to exclude a significant role of the hydroxyl radical, we considered the possible role either of dioxygen, in its ^1^Δ_g_ state, or of the hydroperoxyl radical, HOO. Both are present in appreciable concentrations under normal tropospheric conditions, 10^8^ to 10^9^ molec cm^−3^. The latter was then discarded because the related computed free energy barriers were too high. In contrast, the former was found to open a viable pathway to maleic anhydride. In this case, intervention by two water molecules was found to be essential.

## Data Availability

The data are available upon request to the authors.

## Supplementary Materials

The following supporting information can be downloaded at: https://www.mdpi.com/article/10.3390/molecules28134994/s1. Supplementary Materials for this article include the geometries and energetics of all optimized structures, and some schemes with other mechanisms that have been investigated but ruled out because they were not competitive. Figure S1: active orbitals; Figure S2: displacement vectors for the transition structures; Scheme S1: attempts to form furanones form ketene-enol; Scheme S2: formation of maleic anhydride form ketene-enol initiated by OH; Scheme S3: formation of maleic anhydride form ketene-enol initiated by ^1^O_2_.

## Sample Availability

Not available.

## Appendix A

For the sake of comparison, we report here the characteristics of the first three electronic transitions obtained by TD-DFT [40,41] and EOM-CCSD [26,27,28].

Although the third excited state shows the highest oscillator strength (Table A1), it lays well above the ground state, and its contribution should be negligible because of the scarce sunlight irradiation at ground level at a wavelength below 290–300 nm (see, for instance, Figure 3.32, ([35], p. 80)).

**Table A1:** Characteristics of the first three electronic transitions.

Method	Final State x	λ/nm	f_1x_	Transition ^1^
TD-DFT(M06-2X/cc-pVTZ)	S_1_	430.09	0.0003	n→π^4^
	S_2_	360.83	0.0000	n′→π^4^
	S_3_	222.44	0.3421	π^3^→π^4^
EOM-CCSD/cc-pVTZ	S_1_	377.68	0.0003	n→π^4^
	S_2_	328.94	0.0000	n′→π^4^
	S_3_	204.31	0.3935	π^3^→π^4^

^1^ n and n′ are the in-phase and out-of-phase combinations of in-plane p_O_ orbitals (see p_O_ + p_O_, and p_O_ − p_O_ in Figure 3 in the Section 3).
