# Supplementary material for: Mechanism of the Photochemical Isomerization and Oxidation of 2-Butenedial: A Theoretical Study
Source: Molecules. 2023 Jun 26;28(13):4994. doi: 10.3390/molecules28134994 (PMC10343516; doi:10.3390/molecules28134994)
Supplement: Supplementary file 1 [file molecules-28-04994-s001.zip › molecules-2445226-supplementary.pdf]

Mechanism of the photochemical isomerization and oxidation of 2-butenedial: A Theoretical Study

Andrea Maranzana and Glauco Tonachini

Supplementary Materials

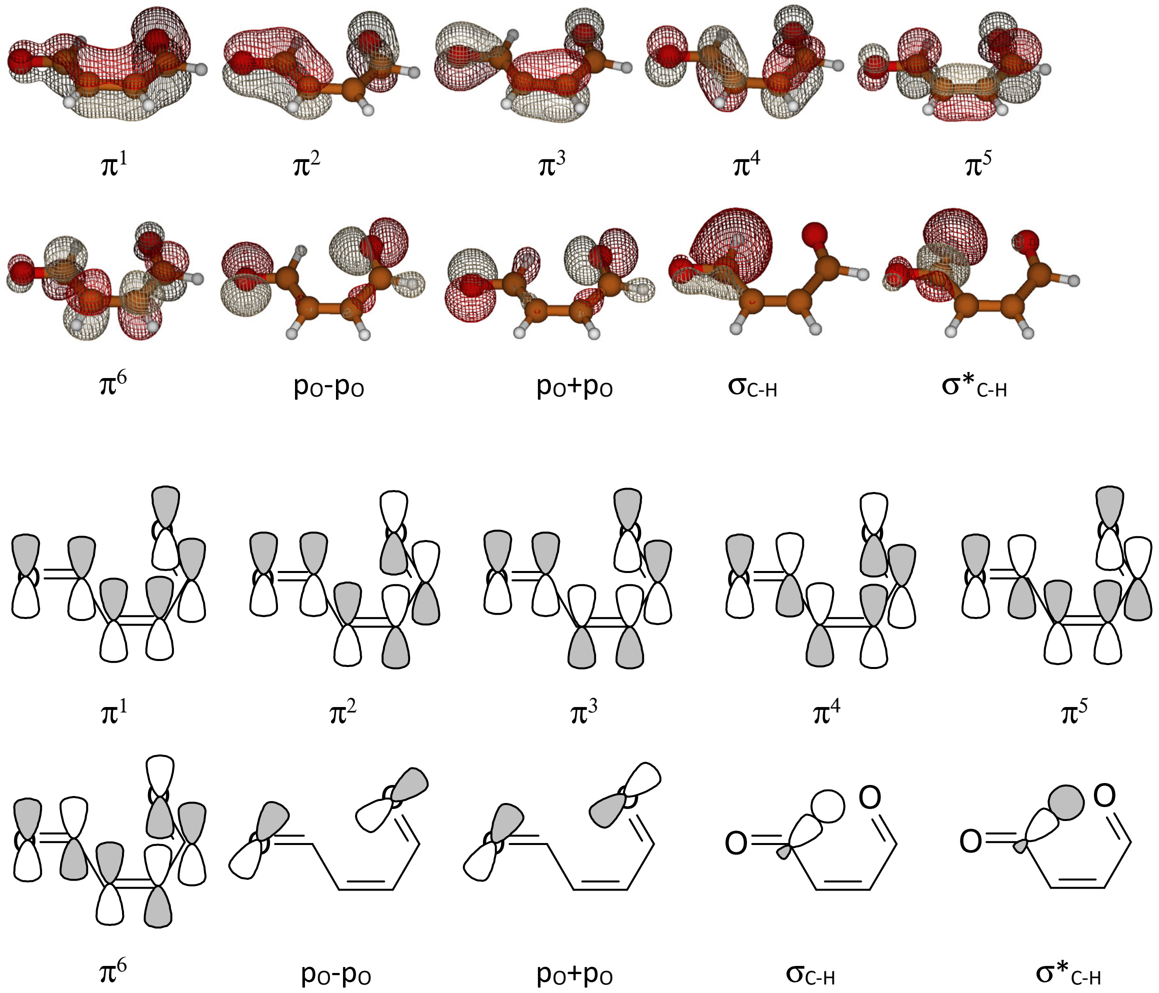

Figure S1. Active orbitals

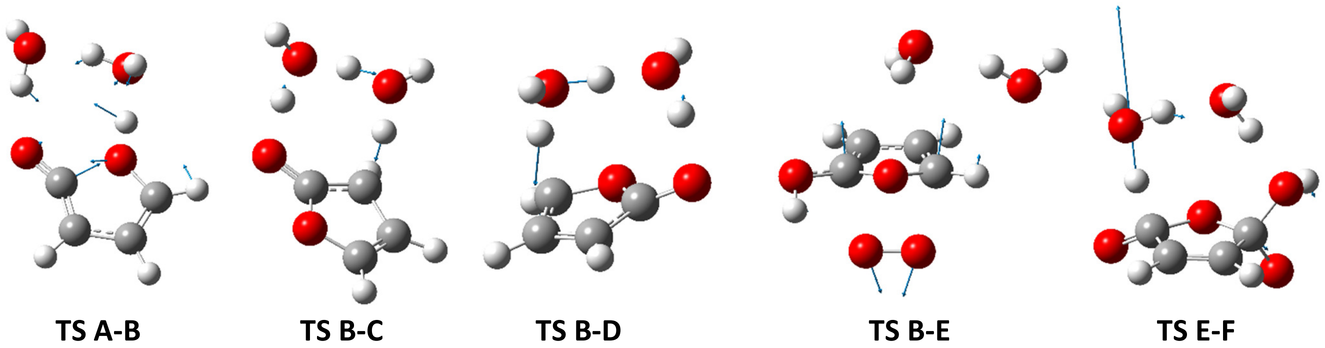

Figure S2. Displacement vectors for the transition structures.

Formation of furanones

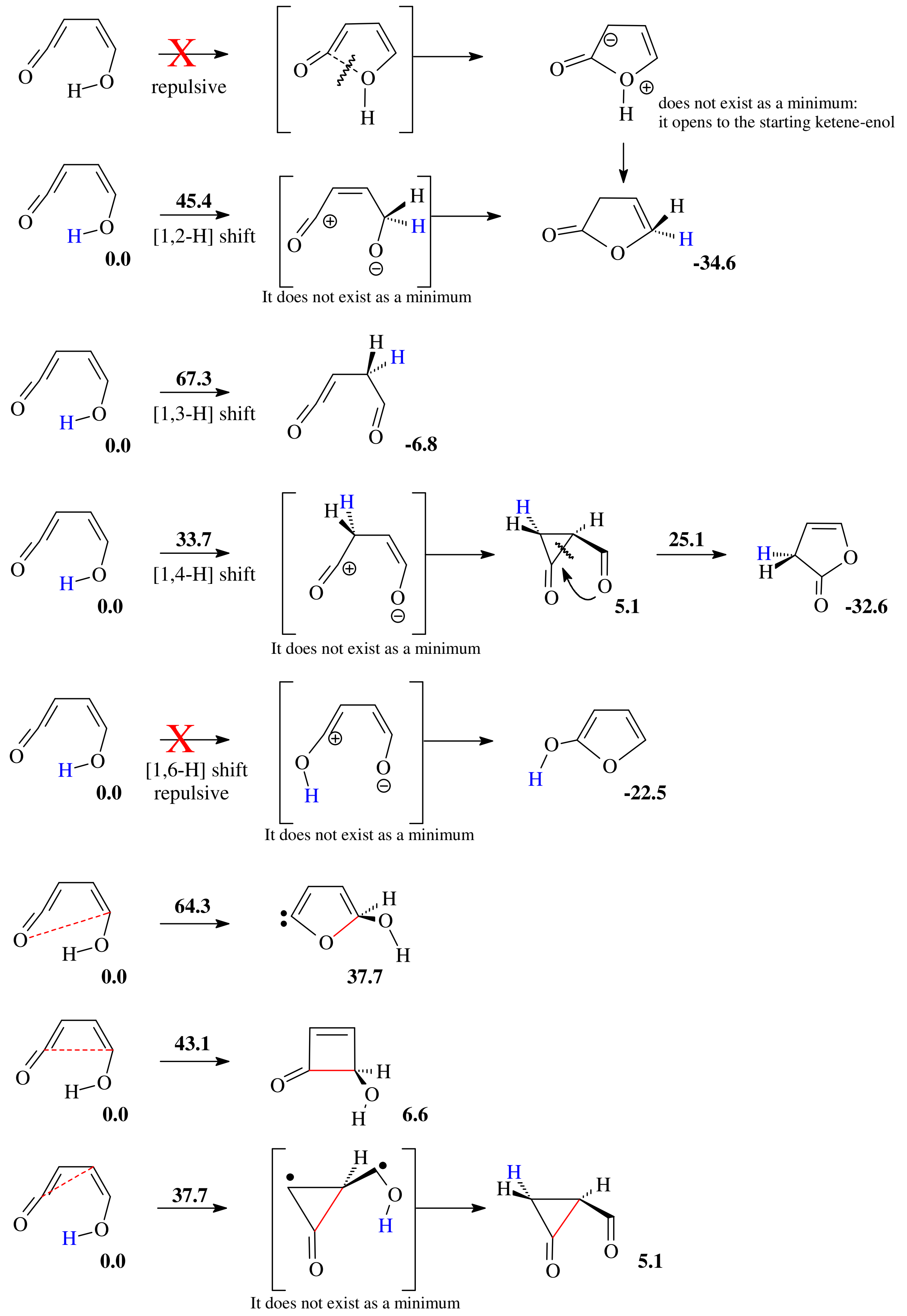

Scheme S1. Attempts to form furanones form ketene-enol, in the ground state. M06-2X/6-31G(d) potential energies in kcal mol^{-1}.

Possible formation of maleic anhydride if the hydroxyl radical had a major role and NOx concentration were substantial.

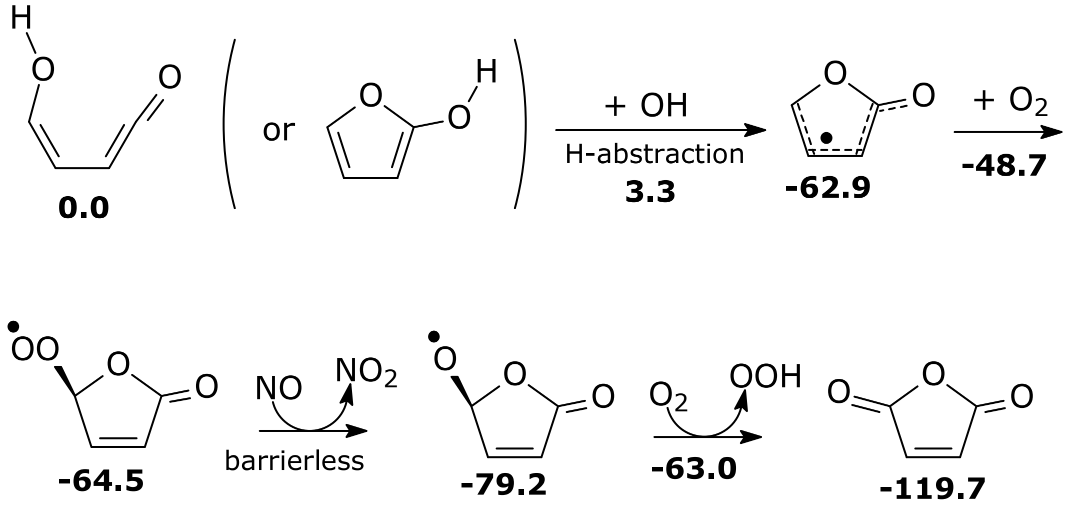

Scheme S2. Formation of maleic anhydride form ketene-enol **A** (or 2-furanol **B**), initiated by OH. ΔG(298K) M06-2X/cc-pVTZ in kcal mol[-1].

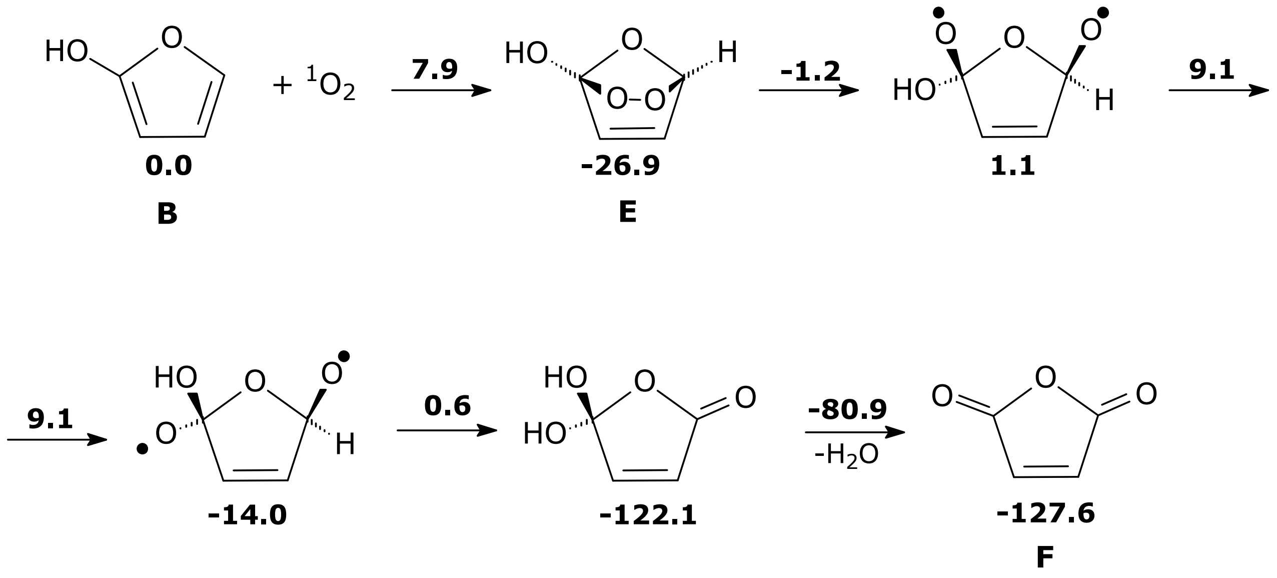

Scheme S3. Formation of maleic anhydride form ketene-enol in the gas phase, initiated by $^1O_2$, without intervention of water molecules. ΔG(298K) M06-2X/cc-pVTZ in kcal mol[-1].

Cartesian coordinates and energies

Formation of furanones without water molecules:

A

Atom	X	Y	Z (Angstrom)
8	2.142733	-0.668503	-0.000002
8	-0.939391	-1.272032	-0.000003
6	1.331917	0.152744	0.000000
6	0.479153	1.159488	0.000004
6	-0.973665	1.052283	-0.000002
6	-1.637058	-0.097448	-0.000001
1	-1.534099	-2.023099	0.000033
1	-1.535363	1.972664	-0.000008
1	-2.716220	-0.156261	-0.000006
1	0.956866	2.128581	0.000012

Energy -305.216208 (Hartree)
Frequencies (cm-1):
103.18 150.41 212.77 269.22 479.16 536.65 591.82 618.85
741.80 828.88 921.98 949.66 1084.24 1150.33 1226.15 1284.52
1413.37 1461.13 1764.24 2238.80 3220.36 3224.50 3257.09 3926.22

TS A–B

Atom	X	Y	Z (Angstrom)
8	-1.967494	0.023086	-0.097644
8	-0.123675	-0.984478	0.136748
6	-0.757545	0.321361	0.077214
6	0.247301	1.235290	0.059178
6	1.485765	0.499095	-0.050225
6	1.264654	-0.824738	-0.073283
1	-1.167272	-1.223046	-0.223980
1	0.120117	2.299514	0.009894
1	2.467468	0.939140	-0.114171
1	1.867994	-1.710525	-0.061880

Energy -305.141098 (Hartree)
Frequencies (cm-1):
1648.25i 220.91 527.04 573.94 616.21 655.67 730.44 742.25
780.17 860.54 918.27 953.99 1015.84 1058.28 1129.86 1184.92
1302.96 1370.13 1609.78 1800.55 2256.02 3267.03 3315.78 3338.36

B

Atom	X	Y	Z (Angstrom)
8	-2.002732	0.089243	0.000031
8	-0.021374	-1.045085	0.000004
6	-0.664845	0.134841	-0.000015
6	0.194892	1.176821	-0.000007
6	1.500686	0.576194	0.000008
6	1.321220	-0.758144	0.000003
1	-2.281408	-0.832205	-0.000160
1	-0.065590	2.218658	-0.000043
1	2.447546	1.087248	0.000003
1	1.980587	-1.605239	-0.000014

Energy -305.243573 (Hartree)
Frequencies (cm-1):
173.63 299.20 430.87 620.57 680.39 697.39 739.22 796.20
896.17 901.56 988.66 1024.38 1106.17 1174.19 1200.81 1281.89
1327.94 1475.05 1600.03 1715.78 3282.28 3308.04 3318.61 3873.39

TS B–C

Atom	X	Y	Z (Angstrom)
8	1.564690	0.169298	0.535997
8	0.215042	-0.985112	-0.642180
6	0.709233	0.259902	-0.393812

```
  6    -0.370324     1.205803    -0.336800
  6    -1.334302     0.418541     0.225032
  6    -0.818697    -0.919208     0.322607
  1     0.606022    -0.485422     1.193709
  1    -1.433901    -1.804649     0.397152
  1    -2.254153     0.750566     0.679872
  1    -0.271295     2.275778    -0.323435
Energy         -305.084168  (Hartree)
Frequencies (cm-1):
 1862.31i    365.84    459.10    608.46    650.39    692.14    761.73    825.63
923.22    941.87    992.17   1026.77   1085.41   1131.52   1144.17   1191.43
1328.70   1385.65   1497.04   1525.64   1721.19   3227.93   3258.11   3300.20
```

C

```
 Atom      X            Y            Z (Angstrom)
  8     2.005502    -0.029346     0.000031
  8     0.049450    -1.131093    -0.000032
  6     0.819609     0.009309    -0.000001
  6    -0.115136     1.207330    -0.000025
  6    -1.464188     0.558967     0.000024
  6    -1.277499    -0.750623     0.000010
  1     0.088972     1.820534     0.878483
  1    -2.415812     1.060710     0.000072
  1    -1.978416    -1.568045     0.000034
  1     0.088926     1.820413    -0.878632
Energy         -305.256241  (Hartree)
Frequencies (cm-1):
   172.08    444.62    501.52    577.39    684.09    740.01    832.18    867.06
950.87    973.60    993.44   1094.34   1138.74   1169.44   1190.48   1290.93
1385.70   1443.96   1710.44   1938.03   3088.16   3124.45   3272.69   3295.97
```

TS B-D

```
 Atom      X            Y            Z (Angstrom)
  8    -2.044741    -0.046791     0.020288
  8    -0.006691    -1.112834    -0.084435
  6    -0.869454     0.111612    -0.036381
  6     0.056643     1.188906    -0.019665
  6     1.348311     0.735562     0.024613
  6     1.434794    -0.653369     0.052393
  1     0.530883    -1.310666     0.871933
  1     2.226373     1.364040     0.040462
  1    -0.274591     2.211824    -0.019849
  1     2.107026    -1.284458    -0.505138
Energy         -305.139832  (Hartree)
Frequencies (cm-1):
 1687.54i    220.73    473.11    527.84    553.23    632.64    677.43    735.19
792.72    819.05    858.12    974.73   1085.78   1097.67   1118.03   1136.87
1278.43   1450.50   1566.01   1921.74   2458.25   3240.78   3263.12   3294.87
```

D

```
 Atom      X            Y            Z (Angstrom)
  8    -2.028810    -0.037266     0.000000
  8    -0.045353    -1.092452     0.000005
  6    -0.838676     0.019065     0.000000
  6     0.055988     1.202992    -0.000003
  6     1.310566     0.779790     0.000005
  6     1.320848    -0.716902    -0.000004
  1     1.807930    -1.129003     0.886016
  1    -0.325354     2.210048     0.000001
  1     2.210465     1.376011     0.000006
  1     1.807913    -1.128982    -0.886043
Energy         -305.259922  (Hartree)
Frequencies (cm-1):
```

206.80 359.82 504.66 683.88 707.87 801.93 840.24 900.98
962.48 988.54 1046.28 1079.84 1152.82 1179.89 1225.46 1362.68
1388.26 1503.74 1693.75 1909.12 3068.66 3105.64 3245.65 3282.68

Formation of furanones with 1 water molecule:

A

Atom	X	Y	Z (Angstrom)
6	-1.446569	0.775397	0.488447
6	-0.112953	1.375604	0.541915
6	0.921176	1.031405	-0.195433
8	1.878749	0.743263	-0.779975
6	-1.776661	-0.309204	-0.209750
8	-0.904326	-1.031969	-0.960123
1	0.073104	2.236807	1.168827
1	-2.218076	1.279624	1.050029
1	-2.802311	-0.653869	-0.253771
1	-0.159191	-1.334336	-0.406940
8	1.116087	-1.736053	0.823582
1	1.957256	-1.429334	0.470882
1	0.915173	-1.140033	1.552023

Energy -381.654788 (Hartree)

Frequencies (cm-1):
30.56 86.42 113.07 142.94 189.42 210.94 270.57 339.45
364.68 471.29 525.70 590.53 625.62 669.57 774.77 850.00
941.78 957.50 1091.55 1149.83 1262.85 1396.26 1413.55 1450.86
1625.38 1729.04 2222.63 3196.50 3214.19 3240.63 3561.71 3838.03
3930.76

TS A–B

Atom	X	Y	Z (Angstrom)
6	-1.177230	-1.254028	-0.077439
8	0.070375	-0.672013	-0.324586
6	-0.038595	0.818437	-0.096580
6	-1.363737	1.002783	0.167948
6	-2.040191	-0.263599	0.178640
8	1.041426	1.413861	-0.169168
8	2.376535	-0.688558	0.165301
1	1.101711	-0.971076	-0.033770
1	2.143363	0.299707	0.052672
1	2.704521	-0.817462	1.059665
1	-1.251985	-2.315929	-0.210524
1	-1.793799	1.966530	0.365876
1	-3.091982	-0.409641	0.358281

Energy -381.634683 (Hartree)

Frequencies (cm-1):
461.39i 78.12 266.53 342.37 439.40 469.44 539.59 568.84
597.71 621.62 702.22 709.99 746.27 828.93 914.02 995.86
1021.58 1048.31 1124.85 1178.29 1202.54 1254.66 1353.13 1403.32
1592.28 1633.83 1836.30 1945.19 2854.36 3271.92 3304.27 3325.37
3903.18

B

Atom	X	Y	Z (Angstrom)
6	-1.497982	0.958164	0.046766
6	-0.182224	0.648956	-0.014249
8	-0.000383	-0.694079	-0.055264
6	-1.251746	-1.265664	-0.020108
6	-2.187407	-0.301618	0.041604
8	0.901991	1.411747	-0.043730
8	2.844270	-0.516415	-0.038342

1	-1.911936	1.948163	0.087073
1	-3.251448	-0.456962	0.079194
1	-1.278791	-2.338400	-0.047811
1	3.346897	-0.677492	0.763325
1	1.704023	0.850445	-0.057358
1	2.140387	-1.174811	-0.049810

Energy -381.685522 (Hartree)
Frequencies (cm-1):
28.87 118.07 154.79 198.36 239.43 296.03 434.40 507.29
613.64 672.98 701.77 704.30 728.62 793.59 896.20 900.63
979.37 1024.99 1113.01 1185.97 1229.54 1300.30 1372.14 1487.30
1606.33 1620.39 1724.01 3281.42 3307.85 3320.87 3516.57 3834.77
3947.64

TS B–C

Atom	X	Y	Z (Angstrom)
6	-1.606534	-0.099648	-0.522374
6	-0.668250	-1.097337	-0.687599
6	0.354172	-0.820549	0.253657
8	-0.116454	0.094473	1.146375
6	-1.196279	0.737604	0.526952
8	1.594702	-0.957019	0.191237
8	1.118941	1.436153	-0.576019
1	-0.575006	-1.808425	-1.488350
1	-2.473373	0.083186	-1.138588
1	-1.825385	1.315566	1.184288
1	1.195184	1.520738	-1.533726
1	1.574636	0.526209	-0.275721
1	0.027776	1.453461	-0.264464

Energy -381.613341 (Hartree)
Frequencies (cm-1):
1074.07i 163.68 315.02 374.42 419.35 464.90 512.64 650.26
690.79 709.74 783.27 820.72 847.74 919.24 944.98 992.90
1050.16 1104.52 1152.08 1194.13 1253.36 1267.63 1347.33 1451.09
1536.03 1616.29 1688.37 1702.84 2231.10 3244.00 3259.60 3294.13
3864.29

C

Atom	X	Y	Z (Angstrom)
6	2.140802	0.120630	0.000109
6	1.458188	1.252651	-0.000014
6	0.002968	0.907234	-0.000137
6	0.038011	-0.606964	-0.000062
8	1.341048	-1.011580	0.000085
8	-0.861078	-1.394200	-0.000114
8	-2.914813	0.558545	0.000053
1	-0.556738	1.253702	0.869627
1	1.879045	2.242739	-0.000018
1	3.198162	-0.080550	0.000226
1	-3.853801	0.368158	0.000649
1	-2.471170	-0.301098	-0.000003
1	-0.556570	1.253625	-0.870040

Energy -381.694476 (Hartree)
Frequencies (cm-1):
25.91 86.35 124.27 171.27 186.61 379.28 443.41 504.46
518.89 579.67 689.55 746.85 835.82 881.64 942.74 968.86
998.23 1091.36 1163.32 1180.33 1186.91 1304.94 1386.92 1423.46
1642.87 1708.78 1900.15 3096.35 3126.57 3274.32 3297.49 3760.72
3948.63

TS B–D

Atom	X	Y	Z (Angstrom)
6	1.439162	1.138271	0.091130

6	0.064149	0.852902	0.490414
6	-0.073105	-0.553349	0.285648
8	1.061341	-1.050264	-0.240603
6	1.969379	-0.009159	-0.335419
8	-1.106083	-1.258635	0.344860
8	-2.296859	0.628038	-0.549497
1	-0.393031	1.313818	1.357318
1	1.943474	2.088038	0.130974
1	2.932282	-0.277802	-0.729117
1	-2.932583	1.079021	0.014135
1	-2.010516	-0.341120	-0.105084
1	-1.204331	1.012948	-0.296947

Energy -381.637076 (Hartree)

Frequencies (cm-1):

1552.63i 111.34 276.24 414.08 469.71 513.69 566.85 605.25
619.95 715.63 728.52 784.89 860.35 889.20 908.98 968.63
1033.43 1100.53 1174.58 1188.55 1273.17 1328.20 1396.46 1441.47
1526.57 1573.84 1649.20 1777.13 2094.23 3192.89 3279.41 3313.14
3893.00

D

Atom	X	Y	Z (Angstrom)
6	-2.184425	-0.113743	-0.015220
6	-1.283421	-1.307187	0.000336
6	-0.021462	-0.903271	0.012511
6	-0.020324	0.577576	0.006280
8	-1.307443	1.002932	-0.011981
8	0.903738	1.342604	0.015786
8	3.054530	-0.532000	0.061633
1	0.897456	-1.467016	0.024019
1	-1.651041	-2.321976	0.001668
1	-2.828467	-0.063342	0.864426
1	3.720586	-0.381266	-0.610598
1	2.523413	0.277333	0.081545
1	-2.810748	-0.072277	-0.908002

Energy -381.698858 (Hartree)

Frequencies (cm-1):

33.24 68.88 128.33 172.19 208.63 326.76 362.75 517.67
585.41 695.30 711.65 806.71 848.55 916.47 964.59 1007.46
1045.94 1085.66 1152.03 1206.45 1224.61 1363.01 1393.60 1500.92
1647.28 1691.42 1872.08 3074.24 3112.89 3246.77 3273.48 3745.53
3944.88

TS C–D

Atom	X	Y	Z (Angstrom)
6	0.806260	-1.047780	-0.611835
6	0.878537	-0.965165	0.759609
6	-0.195624	-0.167859	1.202062
6	-1.082583	0.014825	0.073141
8	-0.391729	-0.504234	-1.036966
8	-2.152770	0.543213	-0.064456
8	1.380061	1.525719	-0.265601
1	-0.488920	0.028494	2.218092
1	1.680382	-1.371232	1.357589
1	1.304665	-1.711449	-1.297394
1	2.175549	1.833209	0.186193
1	1.521697	0.463415	-0.535950
1	0.682589	1.235846	0.469792

Energy -381.604311 (Hartree)

Frequencies (cm-1):

968.42i 131.51 239.16 404.52 443.27 480.38 517.22 522.92
610.67 654.67 716.04 764.00 785.12 850.38 886.74 926.26
1018.83 1107.30 1114.77 1162.94 1220.12 1301.52 1336.72 1446.00

1536.09	1632.82	1756.15	1860.25	2550.29	3240.58	3274.47	3287.07
3855.87							

Formation of furanones with 2 water molecules:

A

Atom	X	Y	Z (Angstrom)
8	0.948718	2.056997	0.243327
8	-0.316058	-0.735348	-0.020283
6	1.583566	1.093340	0.127366
6	2.370044	0.048065	-0.012827
6	1.923639	-1.339393	-0.102642
6	0.642310	-1.691768	-0.094049
1	-1.215974	-1.092046	0.127391
8	-2.034491	1.513567	-0.399319
1	-1.186416	1.079239	-0.557848
1	-1.813677	2.284114	0.128925
8	-2.953826	-0.970620	0.339642
1	-2.908490	-0.009245	0.185560
1	-3.564634	-1.313441	-0.315352
1	0.313928	-2.720721	-0.150118
1	2.681832	-2.101380	-0.180278
1	3.421341	0.295246	-0.052306

Energy -458.101961 (Hartree)

Frequencies (cm-1):

21.14	34.72	83.88	107.53	123.00	166.20	187.44	209.78
227.39	246.48	282.21	305.63	378.81	469.47	499.86	530.52
607.10	622.23	715.80	756.96	808.91	855.02	930.48	955.05
1101.67	1144.72	1255.95	1383.84	1419.27	1453.43	1632.26	1648.25
1758.73	2218.81	3213.15	3223.84	3258.39	3483.34	3638.21	3814.74
3934.20	3935.09						

TS A–B

Atom	X	Y	Z (Angstrom)
8	-0.529914	-1.566459	-0.239557
8	0.485583	0.592357	-0.592500
6	0.514005	-0.993323	-0.067131
6	1.789772	-1.062470	0.386118
6	2.497709	0.184952	0.271778
6	1.726226	1.134397	-0.259466
1	-0.350460	1.102104	-0.232349
8	-2.987307	-0.506498	0.133232
1	-2.181321	-1.052557	0.025428
1	-3.550179	-0.706674	-0.617156
8	-1.580794	1.687229	0.272943
1	-2.245439	0.946848	0.219126
1	-1.543580	1.959835	1.193066
1	1.901903	2.161346	-0.524153
1	2.178374	-1.978853	0.791808
1	3.523886	0.333579	0.563495

Energy -458.087519 (Hartree)

Frequencies (cm-1):

164.01i	25.40	63.23	93.90	202.58	260.19	267.30	301.54
366.90	373.02	418.62	501.16	553.09	581.32	625.02	647.90
683.46	711.64	721.30	854.21	916.41	1011.59	1031.77	1065.03
1112.81	1154.65	1179.08	1343.87	1404.61	1520.16	1641.49	1660.10
1667.96	1916.84	2406.61	3179.94	3267.75	3291.03	3301.89	3531.84
3914.51	3930.55						

B

Atom	X	Y	Z (Angstrom)
8	0.287579	-1.514350	-0.569949
8	-0.341948	0.574395	0.136735
6	-0.688379	-0.693340	-0.224983
6	-2.032926	-0.840096	-0.202621
6	-2.560269	0.432775	0.201821
6	-1.510818	1.249382	0.401896
1	1.339438	1.381088	-0.459963
8	2.528053	-0.922949	0.719815
1	1.111793	-1.339247	-0.046730
1	2.517626	-0.840105	1.675349
8	2.296885	1.508652	-0.507690
1	2.674102	-0.026503	0.363088
1	2.513238	1.546891	-1.441781
1	-3.595795	0.692803	0.335252
1	-2.568178	-1.741248	-0.437066
1	-1.402419	2.268003	0.723888

Energy -458.128841 (Hartree)

Frequencies (cm-1):

28.87 30.52 75.70 141.91 213.56 218.57 240.30 267.16
303.34 321.43 432.95 469.09 532.19 619.17 677.40 700.33
731.20 766.12 804.37 897.55 899.48 953.96 975.81 1025.56
1114.99 1187.82 1228.59 1301.07 1373.89 1458.00 1606.62 1626.35
1642.17 1725.72 3230.83 3281.01 3306.27 3316.83 3593.59 3778.23
3932.32 3935.58

TS B–C

Atom	X	Y	Z (Angstrom)
8	0.553405	-1.547782	0.191732
8	-1.478923	-0.968843	-0.553724
6	-0.375990	-0.715448	0.193925
6	-0.506037	0.573152	0.780216
6	-1.850059	1.012336	0.421317
6	-2.359931	0.080103	-0.387004
1	0.567974	1.234581	-0.099228
8	2.711250	-0.394264	0.145482
1	1.820304	-0.976080	0.248165
1	3.281964	-0.847435	-0.480231
8	1.526631	1.549589	-0.619340
1	2.205017	0.686630	-0.307351
1	1.826483	2.383486	-0.243646
1	-3.288219	-0.006959	-0.921082
1	-2.336049	1.926589	0.715625
1	-0.024274	0.788735	1.723818

Energy -458.092767 (Hartree)

Frequencies (cm-1):

1155.93i 50.17 85.96 119.35 266.22 377.37 404.75 432.69
485.50 544.70 596.29 608.06 655.06 662.73 727.67 752.73
787.41 871.91 887.30 905.70 964.72 1021.10 1108.08 1143.06
1182.55 1203.14 1250.60 1316.21 1401.30 1451.99 1569.85 1614.70
1658.12 1674.95 1727.76 1839.26 2216.75 3216.58 3272.67 3306.74
3881.52 3909.18

C

Atom	X	Y	Z (Angstrom)
8	0.634777	-1.582979	0.532089
8	-0.233152	-0.258623	-1.046761
6	-0.245313	-0.853532	0.182891
6	-1.501420	-0.433412	0.909938
6	-2.159319	0.460732	-0.093470
6	-1.377762	0.516510	-1.156293
1	-1.199229	0.085385	1.819366

8	2.758791	0.043187	-0.462530
1	2.290079	-0.745071	-0.150985
1	2.496218	0.120062	-1.383168
8	0.834314	1.605173	0.900946
1	1.591356	1.197408	0.446525
1	1.203948	2.019193	1.682387
1	-1.466934	1.054383	-2.083944
1	-3.089046	0.986585	0.033119
1	-2.081343	-1.313794	1.188353

Energy -458.137219 (Hartree)

Frequencies (cm-1):

63.45 86.66 101.50 136.59 157.73 177.89 213.85 218.74
236.47 262.26 427.82 437.52 510.90 552.34 587.75 690.60
739.08 777.39 840.58 878.28 942.42 971.78 994.59 1091.84
1161.78 1180.05 1189.25 1298.64 1386.90 1428.58 1647.18 1658.80
1717.47 1900.73 3093.93 3137.80 3276.21 3299.66 3649.83 3761.92
3921.96 3935.65

TS B-D

Atom	X	Y	Z (Angstrom)
8	-0.728427	-1.815938	-0.038150
8	0.309828	-0.168441	1.072962
6	0.254020	-1.058051	0.044556
6	1.341770	-0.796463	-0.828667
6	1.983409	0.322086	-0.342591
6	1.285990	0.794783	0.776288
1	0.212780	1.622028	0.029726
8	-2.384973	0.075363	-0.094052
1	-1.836809	-0.800343	-0.042639
1	-2.849038	0.163021	0.743018
8	-0.775442	1.865704	-0.452561
1	-1.524923	1.056651	-0.272673
1	-0.641690	1.972179	-1.400223
1	1.697142	1.297902	1.637259
1	2.842288	0.815100	-0.772101
1	1.541242	-1.354168	-1.725477

Energy -458.088214 (Hartree)

Frequencies (cm-1):

1009.27i 68.42 110.93 175.22 295.75 343.29 363.48 427.26
451.47 518.12 540.41 579.67 659.41 715.81 735.67 763.51
813.65 862.42 910.77 946.26 991.02 1047.09 1095.43 1102.20
1162.23 1197.49 1286.05 1337.78 1444.04 1456.99 1530.09 1584.75
1640.69 1709.79 1723.85 1793.63 2592.82 3238.73 3253.24 3298.54
3879.15 3903.10

D

Atom	X	Y	Z (Angstrom)
8	-0.587713	-1.736989	-0.345242
8	0.211892	-0.140561	1.002233
6	0.281523	-0.950748	-0.079662
6	1.545654	-0.668768	-0.786094
6	2.140424	0.341030	-0.169017
6	1.300358	0.775220	0.986679
1	0.901575	1.778533	0.829482
8	-2.751874	-0.010973	0.301605
1	-2.225942	-0.798565	0.091441
1	-2.591042	0.128991	1.238024
8	-0.834882	1.686345	-0.891325
1	-1.579497	1.189120	-0.509287
1	-1.149843	2.003002	-1.739030
1	1.848190	-1.209771	-1.665908
1	3.067130	0.824816	-0.436860
1	1.822287	0.720887	1.942542

Energy -458.142888 (Hartree)
Frequencies (cm-1):

52.22	79.54	91.70	148.69	176.98	182.48	210.68	222.04
251.98	281.89	363.40	431.64	514.26	587.73	680.25	714.46
784.90	807.62	843.25	918.12	965.43	978.66	1040.85	1081.40
1148.40	1205.73	1237.97	1368.38	1399.67	1478.16	1650.26	1655.70
1691.64	1864.18	3081.53	3128.71	3248.48	3288.09	3628.20	3732.83
3920.07	3939.45						

Formation of maleic anhydride without water molecules:

Singlet O_2

Atom	X	Y	Z (Angstrom)
8	0.000000	0.000000	0.594632
8	0.000000	0.000000	-0.594632

Energy -150.298750 (Hartree)
Frequencies (cm-1):
1753.05

B

Atom	X	Y	Z (Angstrom)
6	-1.468769	0.642813	-0.000003
6	-1.362997	-0.699590	-0.000007
8	-0.043845	-1.066139	-0.000002
6	0.667212	0.064979	0.000007
6	-0.128411	1.161422	0.000008
8	1.992640	-0.137049	0.000020
1	2.435243	0.715417	-0.000189
1	-2.383409	1.209287	-0.000004
1	-2.071198	-1.506432	-0.000009
1	0.186793	2.189490	0.000024

Energy -305.242255 (Hartree)
Frequencies (cm-1):

223.03	303.35	438.74	621.36	687.54	699.67	741.52	777.95
890.90	902.35	995.29	1028.14	1111.94	1169.69	1206.68	1275.30
1340.23	1506.29	1597.94	1676.65	3281.42	3293.57	3320.30	3903.11

TS B-E

Atom	X	Y	Z (Angstrom)
8	1.267927	-1.314343	-0.111419
6	1.097854	0.556595	0.789668
8	-0.160829	0.214667	1.151668
6	-0.881063	0.288549	0.021570
8	0.114028	-1.463270	-0.588321
6	-0.203110	1.026368	-0.946189
6	1.071856	1.207488	-0.438063
8	-2.154602	-0.058955	0.068757
1	-2.234134	-0.877174	0.576221
1	1.927374	1.626909	-0.936334
1	1.847849	0.494134	1.555622
1	-0.586504	1.257332	-1.922908

Energy -455.522928 (Hartree)
Frequencies (cm-1):

598.57i	157.41	201.38	262.65	314.81	362.60	447.69	534.86
594.52	616.49	706.28	785.38	852.58	893.21	926.48	973.70
1058.04	1119.30	1168.12	1208.21	1292.20	1303.73	1396.14	1496.71
1518.63	1632.60	3290.47	3308.66	3318.73	3821.05		

E

Atom	X	Y	Z (Angstrom)
6	-1.213932	-0.193540	-0.561669
8	0.032357	-0.215750	-1.198522
6	0.742618	0.129638	-0.028096
6	0.031426	1.368477	0.464471
6	-1.233304	1.163615	0.123784
8	2.078248	0.156412	-0.139331
1	2.376750	-0.726334	-0.392924
1	-2.116006	1.718156	0.396110
1	-2.033575	-0.515115	-1.191804
1	0.479274	2.122701	1.089870
8	0.334166	-0.944457	0.865304
8	-1.028181	-1.172272	0.486026

Energy -455.583838 (Hartree)

Frequencies (cm-1):

305.05 323.90 392.71 426.25 461.54 555.19 684.17 702.06
736.18 753.55 843.62 878.78 904.33 949.28 977.15 990.95
1002.09 1052.91 1081.23 1145.72 1278.46 1305.45 1338.13 1366.77
1488.30 1674.54 3204.63 3261.59 3285.45 3829.50

TS E-int1

Atom	X	Y	Z (Angstrom)
6	-0.153246	1.360741	-0.435905
6	1.122346	1.257246	-0.109996
6	1.261894	-0.115493	0.545122
8	-0.004740	-0.328535	1.120225
6	-0.782054	0.052596	0.004340
8	1.416941	-1.091452	-0.437547
8	-2.115231	0.071664	0.242360
8	-0.504237	-0.934886	-0.978843
1	-2.381911	-0.816497	0.512496
1	1.947947	1.904943	-0.354975
1	2.066103	-0.261702	1.268601
1	-0.667651	2.108391	-1.017044

Energy -455.531103 (Hartree)

Frequencies (cm-1):

501.50i 270.05 330.79 383.56 419.76 444.80 518.92 620.69
669.08 696.40 758.06 834.13 864.44 926.53 970.60 991.45
1010.10 1044.36 1068.41 1115.23 1248.41 1277.18 1311.96 1329.22
1448.43 1695.91 3104.18 3257.66 3281.67 3827.42

intermediate1

Atom	X	Y	Z (Angstrom)
6	1.371739	-0.103626	0.490010
8	0.161621	-0.819984	0.489094
6	-0.878901	-0.036664	-0.062571
6	-0.261959	1.309422	-0.318180
6	1.019191	1.272799	-0.010419
8	-1.953198	0.038343	0.806230
1	-2.248316	-0.863851	0.985702
1	1.748200	2.062081	-0.106994
1	1.802037	-0.115329	1.495126
1	-0.843304	2.118222	-0.730707
8	-1.399378	-0.643666	-1.179498
8	2.196075	-0.806282	-0.395347

Energy -455.542077 (Hartree)

Frequencies (cm-1):

77.01 188.79 279.59 353.61 359.92 385.43 485.61 554.69
612.51 691.02 770.66 835.30 863.76 972.94 1002.16 1035.75
1098.68 1117.68 1123.16 1146.84 1200.31 1313.22 1361.72 1433.22
1442.65 1723.77 3071.30 3248.34 3272.61 3825.04

TS int1-int2

```
 Atom      X            Y            Z (Angstrom)
  6     1.096737     1.220327     0.026617
  6     1.410442    -0.220790     0.401911
  8     0.155217    -0.881117     0.379432
  6    -0.847264    -0.011568    -0.001858
  6    -0.193672     1.319556    -0.215457
  8     2.320738    -0.732959    -0.436803
  8    -1.966214    -0.039659     0.834088
  8    -1.667264    -0.484303    -1.027672
  1    -2.468722    -0.594362    -0.123295
  1     1.867637     1.970090    -0.039656
  1     1.825770    -0.301513     1.419403
  1    -0.761960     2.184939    -0.516085
Energy         -455.515655  (Hartree)
Frequencies (cm-1):
 1905.35i     79.92    182.81    329.83    376.86    461.79    500.13    577.43
587.46    635.01    758.88    796.37    857.68    903.76    939.38    986.35
991.08   1050.97   1097.54   1128.35   1165.77   1199.70   1265.72   1332.44
1383.41   1720.91   2274.16   2973.85   3258.76   3281.98
```

intermediate2

```
 Atom      X            Y            Z (Angstrom)
  6     1.044035     1.229216    -0.068728
  6     1.373163    -0.168852     0.436170
  8     0.119895    -0.821277     0.533613
  6    -0.902573    -0.015434     0.022976
  6    -0.245021     1.294575    -0.325303
  8     2.245696    -0.755978    -0.397066
  8    -1.922209     0.076442     0.945121
  8    -1.523606    -0.564238    -1.088499
  1     1.808745     1.975148    -0.209594
  1     1.835073    -0.157388     1.435801
  1    -0.816625     2.123382    -0.711091
  1    -1.803032    -1.457761    -0.851148
Energy         -455.556831  (Hartree)
Frequencies (cm-1):
    71.27    173.64    251.48    337.44    359.58    402.14    498.50    556.04
583.25    688.61    753.10    798.54    875.08    906.37    992.10   1029.70
1087.58   1094.19   1114.51   1128.42   1171.18   1213.18   1294.88   1335.02
1433.02   1722.18   2984.76   3255.36   3279.43   3828.81
```

TS int2-int3

```
 Atom      X            Y            Z (Angstrom)
  6    -0.898288    -0.041923    -0.031341
  6    -0.401402     1.394584    -0.089364
  6     0.912301     1.317064    -0.006746
  6     1.232852    -0.188868     0.000705
  8     0.122479    -0.781892    -0.650970
  8     2.375513    -0.689774    -0.110060
  8    -0.895739    -0.479265     1.316658
  8    -2.135250    -0.249293    -0.548512
  1     1.674208     2.070566     0.099152
  1     0.903005    -0.416851     1.176580
  1    -1.051175     2.254498    -0.095058
  1    -2.334833    -1.191567    -0.477120
Energy         -455.526325  (Hartree)
Frequencies (cm-1):
 1607.18i    261.60    292.23    304.12    356.44    407.14    461.84    505.40
594.86    634.06    682.17    704.44    771.71    825.11    864.58    892.75
953.20    981.53   1044.86   1046.92   1149.42   1268.33   1300.53   1406.46
1432.65   1676.87   1706.80   3260.62   3287.40   3829.23
```

intermediate3

```
 Atom      X            Y            Z (Angstrom)
  6     1.028907     1.254540    -0.054435
  6     1.393964    -0.186621     0.021131
  8     0.233561    -0.910332     0.029056
  6    -0.894522    -0.034217     0.013277
  6    -0.287382     1.349020    -0.058277
  8     2.469370    -0.690898     0.054623
  8    -1.637865    -0.147144     1.153390
  8    -1.697009    -0.371091    -1.067399
  1    -2.116842    -0.983622     1.111298
  1     1.775186     2.030175    -0.093805
  1    -0.915708     2.224719    -0.095843
  1    -1.132894    -0.611882    -1.809191
Energy         -455.733132  (Hartree)
Frequencies (cm-1):
   115.18    198.08    248.00    301.84    403.10    412.33    459.86    567.29
576.18    686.69    723.88    747.50    858.92    872.47    959.41   1007.53
1038.67   1086.23   1138.75   1174.05   1242.76   1336.73   1376.18   1461.02
1714.47   1927.23   3258.25   3284.40   3838.05   3873.71
```

TS int3-F

```
 Atom      X            Y            Z (Angstrom)
  6     0.818163     0.041850    -0.239821
  6     0.208746     1.380964     0.056519
  6    -1.101563     1.239467     0.149978
  6    -1.425638    -0.200870    -0.029916
  8    -0.244126    -0.865755    -0.280811
  8    -2.471437    -0.757181     0.019879
  8     1.804397    -0.128618    -1.040484
  8     1.706797    -0.404485     1.099242
  1     2.308744    -0.516627     0.096621
  1    -1.865934     1.976758     0.329423
  1     0.817983     2.266898     0.130961
  1     1.375900    -1.247185     1.439822
Energy         -455.662271  (Hartree)
Frequencies (cm-1):
 1656.36i    120.04    191.41    301.38    384.67    398.18    484.45    551.13
580.74    674.53    707.54    763.00    832.41    873.34    893.98    929.32
958.08   1005.43   1076.46   1107.08   1292.15   1333.16   1342.80   1560.43
1704.92   1939.62   2076.93   3265.84   3288.18   3818.20
```

F

```
 Atom      X            Y            Z (Angstrom)
  6     0.661714     1.255844     0.000000
  6     1.121775    -0.162477    -0.000000
  8    -0.000000    -0.964313     0.000000
  6    -0.661714     1.255844    -0.000000
  6    -1.121775    -0.162476    -0.000000
  8    -2.222913    -0.597813     0.000000
  1    -1.356309     2.079547     0.000000
  1     1.356310     2.079547     0.000000
  8     2.222913    -0.597813     0.000000
Energy         -379.295342  (Hartree)
Frequencies (cm-1):
   166.40    271.13    413.70    566.74    650.47    653.68    715.46    797.77
883.09    893.25    961.60   1014.93   1076.54   1089.83   1311.70   1336.73
1693.55   1910.43   1978.87   3265.64   3286.84
```

Formation of maleic anhydride with 2 water molecules:

Singlet O_2

```
 Atom      X            Y            Z (Angstrom)
  8     0.000000     0.000000     0.594632
  8     0.000000     0.000000    -0.594632
Energy         -150.298750  (Hartree)
Frequencies (cm-1):
  1753.05
```

B

```
 Atom      X            Y            Z (Angstrom)
  6    -1.277937    -0.368040    -0.230405
  6    -1.650436     0.646763     0.582602
  6    -0.703042     1.697852     0.310284
  6     0.146793     1.231612    -0.628465
  8    -0.206006    -0.047482    -0.969317
  8    -1.770620    -1.601892    -0.393163
  8     2.839992    -0.219541    -0.695931
  8     1.254301    -0.668482     1.674274
  1    -2.481282     0.649382     1.263090
  1    -1.290483    -2.033488    -1.108648
  1     1.026437     1.608555    -1.114029
  1     0.611924    -1.369572     1.806762
  1     0.724684     0.128292     1.546862
  1     3.724516    -0.085671    -0.351548
  1     2.323868    -0.521788     0.065275
  1    -0.673269     2.674351     0.761230
Energy         -458.116742  (Hartree)
Frequencies (cm-1):
   39.66     51.81     96.77    107.22    134.93    151.82    175.28    192.55
201.91    256.98    302.23    323.29    432.36    454.20    624.18    665.85
692.92    699.14    768.50    801.37    900.95    906.72    993.63   1022.38
1108.01   1177.00   1203.42   1281.51   1328.21   1476.02   1591.48   1617.55
1649.73   1712.35   3281.56   3305.53   3331.50   3750.37   3817.29   3858.71
3933.47   3938.48
```

TS B-E

```
 Atom      X            Y            Z (Angstrom)
  6    -0.304258    -0.631286     1.052562
  6     0.610398     0.376370     1.326274
  6     1.079576     0.791456     0.082359
  8     0.332436     0.261666    -0.903736
  6    -0.335670    -0.769232    -0.329966
  8     1.889781     1.792593    -0.221465
  8     2.295951    -0.914423    -0.083670
  8     1.469072    -1.828057    -0.325747
  8    -3.259433    -1.041880    -0.157135
  8    -2.358862     1.689805    -0.086696
  1     2.409463     1.542940    -0.997268
  1    -0.817946    -1.277170     1.741547
  1    -0.976054    -1.351551    -0.965412
  1     1.018699     0.688012     2.270361
  1    -3.133614    -0.081464    -0.150362
  1    -4.207441    -1.181024    -0.178478
  1    -1.841849     1.838585    -0.883148
  1    -1.703084     1.540180     0.602976
Energy         -608.395070  (Hartree)
Frequencies (cm-1):
  580.96i    24.61     42.10     58.44    111.94    128.90    159.11    163.94
195.95    201.64    204.64    212.72    267.68    293.92    314.04    361.60
437.95    455.08    531.13    595.79    614.59    652.15    705.59    792.16
870.66    891.89    924.78    971.55   1055.99   1119.15   1169.17   1200.86
```

1288.83	1303.10	1387.19	1495.21	1508.10	1627.15	1629.12	1645.38
3293.89	3307.10	3326.32	3735.90	3814.07	3837.26	3932.46	3941.56

E

Atom	X	Y	Z (Angstrom)
8	1.033648	-1.717263	-0.456810
6	-0.173078	-0.938026	-0.344751
8	0.248851	0.300198	-0.878229
6	1.292740	0.455328	0.076033
8	2.051511	-0.751660	-0.160899
6	-0.356749	-0.627470	1.130666
6	0.583825	0.269252	1.397735
8	2.068984	1.536691	-0.088645
8	-3.130096	-0.832573	-0.021125
8	-2.176536	1.799740	-0.305602
1	2.532125	1.451252	-0.932173
1	-1.037802	-1.146746	1.783025
1	-0.972578	-1.411795	-0.897417
1	0.926475	0.676140	2.334608
1	-2.979799	0.121809	-0.123635
1	-4.008487	-0.997728	-0.366903
1	-1.312233	1.513963	-0.632651
1	-1.999026	2.157525	0.567532

Energy -608.462134 (Hartree)

Frequencies (cm-1):

34.28	51.53	80.13	132.08	143.72	169.91	211.46	222.41
283.93	308.40	337.29	369.42	396.45	433.85	463.70	555.36
572.49	686.90	706.42	729.50	742.64	757.67	832.91	877.87
912.88	955.44	986.96	1000.36	1008.31	1042.80	1079.55	1131.63
1267.88	1314.62	1335.49	1358.05	1483.11	1639.13	1645.78	1672.51
3232.68	3266.58	3292.20	3667.73	3776.07	3822.80	3926.43	3939.48

TS E-F

Atom	X	Y	Z (Angstrom)
6	-1.279200	-0.428015	-0.085686
6	-1.041954	0.005304	1.338103
6	-0.223130	1.042351	1.357022
6	0.205747	1.383772	-0.039435
8	-0.471015	0.417240	-0.872793
8	0.382000	2.546502	-0.476736
8	-0.924183	-1.779065	-0.255938
8	-2.506969	-0.201011	-0.595895
1	-1.275151	-2.043510	-1.116543
1	0.111981	1.621888	2.202353
1	1.414097	0.911196	-0.099053
1	-1.548456	-0.467842	2.164591
8	2.644763	0.279766	-0.197177
1	2.406852	-0.686797	-0.102643
1	2.958617	0.422316	-1.096385
8	1.779043	-2.151469	0.109117
1	0.811484	-2.097109	-0.014399
1	1.922682	-2.576312	0.957433

Energy -608.428119 (Hartree)

Frequencies (cm-1):

1452.69i	37.37	68.38	83.40	122.44	184.45	224.27	253.25
312.83	323.33	345.31	365.45	384.73	431.98	462.38	474.56
508.87	555.32	592.74	620.82	692.51	737.18	756.74	822.25
835.96	875.16	976.11	988.43	1001.28	1027.53	1123.83	1138.03
1180.93	1209.21	1305.15	1328.38	1434.97	1537.24	1626.10	1649.07
1699.73	3112.95	3248.13	3272.11	3558.53	3818.20	3881.87	3927.16

F

```
 Atom       X             Y             Z (Angstrom)
  6     0.732436     1.129604     0.370587
  8     0.308939     0.675887    -0.854766
  6    -1.075360     0.585626    -0.836627
  6    -1.532678     1.040410     0.504579
  6    -0.463652     1.377158     1.209445
  8    -1.688744     0.215111    -1.778847
  8     1.888932     1.273854     0.619933
  8    -2.196399    -1.717729     0.608611
  8     0.616313    -1.686056     0.892595
  8     2.719510    -1.112649    -0.817181
  1    -2.441261    -1.941831    -0.291885
  1    -0.386078     1.735591     2.221723
  1     2.812216    -0.222317    -0.451238
  1    -2.573596     1.032844     0.775880
  1     1.357839    -1.633920     0.259257
  1     2.350681    -0.972973    -1.693625
  1    -1.238616    -1.860279     0.656661
  1     0.965946    -2.121259     1.672550
Energy         -608.618856  (Hartree)
Frequencies (cm-1):
    35.49     58.99     64.39    104.20    117.78    142.48    154.75    174.49
186.19    208.36    224.62    256.20    279.06    293.23    295.79    380.52
420.17    468.83    533.31    574.60    643.29    652.98    669.75    714.72
784.88    815.69    872.99    900.83    966.44    994.70   1079.19   1098.30
1320.95   1339.54   1645.34   1649.20   1660.45   1690.82   1889.51   1968.82
3279.67   3305.02   3561.15   3722.21   3789.08   3917.21   3925.29   3929.71
```

Formation of maleic anhydride with OH:

Triplet O_2

```
 Atom       X             Y             Z (Angstrom)
  8     0.000000     0.000000     0.594932
  8     0.000000     0.000000    -0.594932
Energy         -150.321735  (Hartree)
Frequencies (cm-1):
  1758.25
```

OH

```
 Atom       X             Y             Z (Angstrom)
  8     0.000000     0.000000     0.107936
  1     0.000000     0.000000    -0.863486
Energy          -75.730487  (Hartree)
Frequencies (cm-1):
  3776.00
```

OOH

```
 Atom       X             Y             Z (Angstrom)
  8     0.055050    -0.600572     0.000000
  8     0.055050     0.708417     0.000000
  1    -0.880808    -0.862760     0.000000
Energy         -150.904168  (Hartree)
Frequencies (cm-1):
  1254.95   1459.86   3700.76
```

NO

```
 Atom       X             Y             Z (Angstrom)
  7     0.000000     0.000000    -0.606661
  8     0.000000     0.000000     0.530829
Energy         -129.891119  (Hartree)
Frequencies (cm-1):   2074.35
```

NO_2

```
 Atom       X            Y            Z (Angstrom)
  7     0.000000     0.000000     0.315041
  8     0.000000     1.090464    -0.137830
  8     0.000000    -1.090464    -0.137830
Energy         -205.070765  (Hartree)
Frequencies (cm-1):
   784.74   1470.98   1786.94
```

A

```
 Atom       X            Y            Z (Angstrom)
  6    -0.973626     1.052301    -0.000005
  6    -1.637087    -0.097394    -0.000005
  8    -0.939506    -1.272029     0.000004
  6     0.479191     1.159458     0.000009
  6     1.331959     0.152714     0.000000
  8     2.142786    -0.668537    -0.000007
  1     0.956936     2.128539     0.000024
  1    -1.535291     1.972703    -0.000014
  1    -2.716254    -0.156131    -0.000014
  1    -1.534250    -2.023064     0.000026
Energy         -305.216208  (Hartree)
Frequencies (cm-1):
   103.19    150.40    212.64    269.23    479.16    536.67    591.83    618.86
741.80    828.88    921.99    949.66   1084.24   1150.33   1226.14   1284.51
1413.37   1461.13   1764.24   2238.73   3220.35   3224.48   3257.08   3926.26
```

TS Habs

```
 Atom       X            Y            Z (Angstrom)
  6     0.557250     1.568910    -0.140568
  6     1.662833     0.660434    -0.355195
  6     1.655180    -0.637445    -0.090583
  8     1.742891    -1.766513     0.113517
  6    -0.642401     1.189639     0.315519
  8    -0.849070    -0.094688     0.655690
  8    -2.980889    -0.802656    -0.389990
  1    -2.763535    -0.557121    -1.302893
  1     2.598291     1.013005    -0.764925
  1     0.729434     2.613488    -0.348120
  1    -1.459505     1.884984     0.461185
  1    -1.805315    -0.332719     0.545979
Energy         -380.952265  (Hartree)
Frequencies (cm-1):
 1314.23i     30.81     66.21    133.99    155.69    193.97    254.21    293.42
479.45    486.79    536.80    596.27    624.70    776.07    842.91    936.07
961.85   1099.06   1155.42   1231.46   1262.40   1409.60   1463.20   1627.73
2179.90   2693.58   3200.45   3225.85   3250.37   3790.90
```

rad

```
 Atom       X            Y            Z (Angstrom)
  6    -1.403051     0.650773     0.000003
  6    -1.265611    -0.727757    -0.000001
  8     0.013077    -1.105380     0.000000
  6    -0.126744     1.173749    -0.000001
  6     0.804700     0.069171     0.000000
  8     1.999578    -0.003141    -0.000001
  1     0.186755     2.202361     0.000004
  1    -2.339416     1.181160    -0.000010
  1    -2.004342    -1.510970     0.000001
Energy         -304.625857  (Hartree)
Frequencies (cm-1):
   259.76    500.25    534.97    689.70    710.48    764.34    803.63    819.06
884.23    924.02   1046.87   1101.69   1123.54   1225.99   1367.51   1423.56
```

```
1521.12   1816.71   3273.46   3287.61   3300.91
```

TS rad+O_2

```
 Atom      X             Y            Z (Angstrom)
  6     0.328076     1.483335    -0.377870
  6     0.617116     0.797598     0.836693
  8    -0.339820    -0.078000     1.129159
  6    -0.739138     0.866130    -0.924192
  6    -1.219145    -0.146721     0.020768
  8    -2.143910    -0.890219     0.014849
  1    -1.220789     1.044216    -1.869553
  1     0.936725     2.267769    -0.794010
  1     1.276822     1.084612     1.637652
  8     1.910802    -0.553626    -0.001533
  8     1.208651    -1.277986    -0.680785
Energy         -454.943896  (Hartree)
Frequencies (cm-1):
  494.09i    114.51    141.45    215.24    302.32    404.78    500.63    569.93
703.52    716.51    808.69    818.69    864.87    874.86    966.27   1050.92
1098.07   1118.70   1228.59   1367.87   1407.33   1514.71   1591.40   1903.12
3270.95   3282.41   3298.21
```

peroxyl rad

```
 Atom      X             Y            Z (Angstrom)
  6    -0.112795     1.488161     0.125162
  6     0.677306     0.254961     0.459114
  8    -0.216010    -0.812590     0.352510
  6    -1.352395     1.129427    -0.156277
  6    -1.455168    -0.350001    -0.026959
  8    -2.385786    -1.065762    -0.187919
  1    -2.192933     1.737120    -0.445817
  1     0.327921     2.472242     0.116716
  1     1.153990     0.243819     1.437703
  8     1.716639     0.110592    -0.518410
  8     2.656324    -0.680798    -0.085535
Energy         -454.971637  (Hartree)
Frequencies (cm-1):
    71.48    124.05    214.15    351.65    460.01    500.73    565.91    702.93
717.64    825.28    856.52    903.46    999.24   1017.98   1043.63   1089.97
1146.89   1179.57   1258.75   1340.68   1378.92   1384.50   1707.27   1939.09
3134.34   3258.41   3287.89
```

oxyl radical

```
 Atom      X             Y            Z (Angstrom)
  6     0.705035     1.221260     0.126383
  6     1.115681    -0.227292     0.356220
  8    -0.090191    -0.967481     0.244031
  6    -0.595142     1.255147    -0.095798
  6    -1.134820    -0.131864    -0.021423
  8    -2.255283    -0.505896    -0.149527
  1    -1.226500     2.103830    -0.300359
  1     1.427965     2.020555     0.136640
  1     1.516564    -0.378143     1.371012
  8     2.062655    -0.582842    -0.519452
Energy         -379.813215  (Hartree)
Frequencies (cm-1):
   127.07    230.81    388.44    489.11    554.86    696.55    707.15    779.88
849.54    897.32    920.73   1002.25   1061.59   1096.26   1122.70   1168.71
1197.89   1321.01   1350.98   1699.20   1925.01   2983.75   3260.91   3283.52
```

TS Habs by O_2

```
 Atom      X             Y            Z (Angstrom)
  6     1.831225    -0.409043    -0.028475
  8     0.629426    -0.714006     0.546636
  6    -0.263289     0.375444     0.378213
  6     0.532984     1.451215    -0.319386
  6     1.747727     0.981790    -0.551370
  8    -1.141117     0.584375     1.246546
  8     2.743878    -1.169990    -0.070276
  1     2.588275     1.466800    -1.019514
  1     0.103347     2.414072    -0.544325
  1    -1.019946    -0.008964    -0.553420
  8    -2.306592    -0.537331    -1.012227
  8    -3.021040    -0.446591    -0.055259
Energy         -530.121399  (Hartree)
Frequencies (cm-1):
 1335.42i     60.70     96.13    139.83    184.88    232.18    321.54    402.33
522.96    541.13    600.71    684.23    724.41    799.24    854.71    884.31
921.30   1002.30   1049.45   1064.98   1088.12   1231.15   1254.23   1332.22
1615.48   1686.12   1687.83   1920.07   3259.53   3282.07
```

Anhydride

```
 Atom      X             Y            Z (Angstrom)
  6     0.661714     1.255844     0.000000
  6     1.121775    -0.162477    -0.000000
  8    -0.000000    -0.964313     0.000000
  6    -0.661714     1.255844    -0.000000
  6    -1.121775    -0.162476    -0.000000
  8    -2.222913    -0.597813     0.000000
  1    -1.356309     2.079547     0.000000
  1     1.356310     2.079547     0.000000
  8     2.222913    -0.597813     0.000000
Energy         -379.295342  (Hartree)
Frequencies (cm-1):
   166.40    271.13    413.70    566.74    650.47    653.68    715.46    797.77
883.09    893.25    961.60   1014.93   1076.54   1089.83   1311.70   1336.73
1693.55   1910.43   1978.87   3265.64   3286.84
```

S1 – First excited state

2-butenedial

```
 Atom      X             Y            Z (Angstrom)
 C    -1.101424     0.000000    -0.863990
 C    -0.063582     0.000000    -1.869102
 O     1.134633     0.000000    -1.633114
 C    -0.910054     0.000000     0.547255
 C     0.288818     0.000000     1.185044
 O     0.398237     0.000000     2.528802
 H    -1.796521     0.000000     1.156906
 H     1.262501     0.000000     0.679930
 H    -2.113111     0.000000    -1.225175
 H    -0.399141     0.000000    -2.903409
Energy        -303.48573585   (Hartree)
Frequencies (cm-1):
    178.99   188.44   222.79   337.36   408.74   513.74   677.56   742.47
874.77   970.42   985.75  1002.91  1140.09  1225.72  1299.45  1406.42  1538.34
1562.70  1651.74  1750.53  3129.72  3231.92  3353.87  3380.59
```

TS ring closure

Atom	X	Y	Z (Angstrom)
C	-0.431143	0.182898	-0.927672
C	-0.139350	1.267949	0.048409
O	0.419340	-0.136722	-1.823221
O	-0.158124	-1.262794	0.257420
C	0.178821	0.790334	1.316513
C	0.131307	-0.583614	1.394459
H	0.202194	-1.209476	2.260504
H	0.411327	1.407992	2.161605
H	-0.161801	2.299171	-0.242309
H	-1.495469	-0.034863	-1.152629

Energy -303.4393962 (Hartree)

Frequencies (cm-1):

848.56i 163.24 315.71 438.30 515.82 563.77 650.23 690.66 879.60
899.89 903.40 1022.71 1096.03 1153.92 1255.44 1351.73 1405.87 1460.95
1528.50 1614.01 2977.08 3402.70 3415.38 3434.27

Cyclic intermediate – conical intersection

Atom	X	Y	Z (Angstrom)
C	0.077691	-0.328755	0.809312
C	1.222892	-0.091199	-0.130555
O	-0.013548	0.306936	1.924862
O	-1.160742	-0.106883	-0.163730
C	0.743906	0.115105	-1.396605
C	-0.660826	0.126266	-1.355400
H	-1.356979	0.274385	-2.154275
H	1.324797	0.271127	-2.283106
H	2.242601	-0.163577	0.186293
H	-0.058736	-1.429384	1.001604

Energy -303.45989428 (Hartree)

TS H-transfer

Atom	X	Y	Z (Angstrom)
C	-1.102687	0.000000	-0.977294
C	0.079520	0.000000	-1.707912
O	1.264818	0.000000	-1.185807
C	-1.114660	0.000000	0.419966
C	0.130398	0.000000	1.097464
O	0.429289	0.000000	2.261676
H	-2.026338	0.000000	0.986640
H	-2.025941	0.000000	-1.523586
H	0.072752	0.000000	-2.784030
H	1.009655	0.000000	0.158990

Energy -303.4755490 (Hartree)

Frequencies (cm-1):

1292.30i 222.43 388.34 421.79 521.96 553.54 558.71 835.47
873.42 881.52 948.00 1048.81 1173.78 1188.93 1235.19 1373.81 1503.89
1567.31 1604.79 1724.95 1852.36 3361.51 3391.66 3407.55

Ketene-enol – conical intersection

Atom	X	Y	Z (Angstrom)
C	-1.115614	0.000000	-0.859220
C	-0.109487	0.000000	-1.792514
O	1.209426	0.000000	-1.569061
C	-1.014595	0.000000	0.549742
C	0.210815	0.000000	1.264909
O	0.500482	0.000000	2.423036
H	-1.914927	0.000000	1.138103
H	-2.110404	0.000000	-1.265301

```
 H    -0.332155     0.000000    -2.840164
 H     1.392429     0.000000    -0.613104
Energy    -303.5073118        (Hartree)
```

Ketene-enol - ground state

```
 Atom      X            Y            Z (Angstrom)
 C    -1.081513    -0.219250    -0.800812
 C    -0.079280    -0.283169    -1.695876
 O     1.161787     0.235210    -1.571197
 C    -1.002475     0.420317     0.530998
 C    -0.093464     0.062805     1.424258
 O     0.705014    -0.251878     2.206972
 H    -1.721324     1.150251     0.850996
 H    -2.018979    -0.666311    -1.074956
 H    -0.195748    -0.791260    -2.632416
 H     1.195724     0.801849    -0.783594
Energy     -303.55904586      (Hartree)
```

S2 – Second excited state

2-butenedial

```
 Atom      X            Y            Z (Angstrom)
 C    -1.078306     0.000000    -0.851517
 C    -0.145095     0.000000    -1.877670
 O     1.136337     0.000000    -1.722350
 C    -0.868093     0.000000     0.566390
 C     0.338972     0.000000     1.254911
 O     0.369405     0.000000     2.539508
 H    -1.752512     0.000000     1.177665
 H     1.315675     0.000000     0.747957
 H    -2.102899     0.000000    -1.175004
 H    -0.477722     0.000000    -2.902935
Energy       -303.47017495    (Hartree)
Frequencies (cm-1):
    122.12   182.16   208.51   337.11   469.44   525.29   746.68   823.95
859.09   870.81   918.70  1029.31  1204.76  1255.50  1339.16  1377.31  1505.03
1515.14  1597.71  3083.19  3323.25  3360.61  3378.20  4353.57
```

2-butenedial - conical intersection

```
 Atom      X            Y            Z (Angstrom)
 C    -0.992252     0.000000    -0.738913
 C    -0.270230     0.000000    -1.926573
 O     0.972483     0.000000    -2.206733
 C    -0.647916     0.000000     0.664467
 C     0.507477     0.000000     1.442007
 O     0.264192     0.000000     2.708012
 H    -1.525846     0.000000     1.295455
 H     1.555589     0.000000     1.139570
 H    -2.054079     0.000000    -0.915924
 H    -0.888131     0.000000    -2.814688
Energy      -303.47502427     (Hartree)
```

TS H-transfer

```
 Atom      X            Y            Z (Angstrom)
 C    -1.073623     0.000000    -1.048246
 C     0.074165     0.000000    -1.741526
 O     1.297981     0.000000    -1.155462
 C    -1.101879     0.000000     0.413539
```

```
 C     0.053049     0.000000     1.089125
 O     0.420065     0.000000     2.314591
 H    -2.039748     0.000000     0.934354
 H    -1.997687     0.000000    -1.592140
 H     0.158700     0.000000    -2.811469
 H     1.015745     0.000000     0.407644
Energy        -303.43373387   (Hartree)
Frequencies (cm-1):
  2080.73i   92.86   279.87   280.36   464.96  518.14   581.56   587.42   647.38
862.01   899.11  1042.48  1121.18  1142.46  1246.68  1378.09  1482.21  1504.71
1629.05  1716.62  1829.96  3363.95  3389.94  3407.36
```

TS ring closure

```
 Atom      X             Y             Z (Angstrom)
 C    -0.406989     0.144630    -0.839484
 C    -0.149331     1.239922     0.133432
 O     0.394266    -0.041214    -1.861540
 O    -0.147973    -1.191641     0.152777
 C     0.188026     0.727916     1.374679
 C     0.130044    -0.656444     1.340762
 H     0.217466    -1.350808     2.151046
 H     0.401356     1.297425     2.257288
 H    -0.209577     2.274275    -0.138003
 H    -1.479663    -0.001876    -1.091167
Energy        -303.44696585   (Hartree)
Frequencies (cm-1):
   114.64i    78.79   387.62   481.76   531.83   594.26   750.17   889.64
897.34   997.62  1058.23  1135.40  1175.01  1299.25  1362.71  1449.41  1458.03
1544.35  1641.81  2031.32  2950.94  3410.75  3422.60  3441.01
```
